# Patients, primary care, and policy: Agent-based simulation modeling for health care decision support

**DOI:** 10.1007/s10729-021-09556-2

**Published:** 2021-05-25

**Authors:** Martin Comis, Catherine Cleophas, Christina Büsing

**Affiliations:** 1 Lehrstuhl II für Mathematik, RWTH Aachen University, Pontdriesch 10–12, 52062 Aachen, Germany; 2 Working Group Service Analytics, Christian-Albrechts-Universität zu Kiel, Westring 425, 24118 Kiel, Germany

**Keywords:** Hybrid simulation, Agent-based modeling, Discrete-event simulation, Primary care, Decision support, Operations research

## Abstract

Primary care systems are a cornerstone of universally accessible health care. The planning, analysis, and adaptation of primary care systems is a highly non-trivial problem due to the systems’ inherent complexity, unforeseen future events, and scarcity of data. To support the search for solutions, this paper introduces the hybrid agent-based simulation model SiM-Care. SiM-Care models and tracks the micro-interactions of patients and primary care physicians on an individual level. At the same time, it models the progression of time via the discrete-event paradigm. Thereby, it enables modelers to analyze multiple key indicators such as patient waiting times and physician utilization to assess and compare primary care systems. Moreover, SiM-Care can evaluate changes in the infrastructure, patient behavior, and service design. To showcase SiM-Care and its validation through expert input and empirical data, we present a case study for a primary care system in Germany. Specifically, we study the immanent implications of demographic change on rural primary care and investigate the effects of an aging population and a decrease in the number of physicians, as well as their combined effects.

## Highlights

- We present the hybrid agent-based simulation model SiM-Care, which aims to serve as a decision support tool for the analysis of the quality of primary care systems
- Assessments are based on multiple key performance indicators such as patient waiting times and physicians’ utilization
- Effects of interventions such as the use of mobile medical units or centralized appointment systems can be quantified and validated before an actual action is taken
- The simulation model is very generic and can be easily adapted to individual needs and regional specifics
- A case study demonstrates the application of SiM-Care for the analysis of a rural primary care system in Germany

## Introduction

Primary care systems are the foundation of accessible health services. Following the definition of the American Academy of Family Physicians [5], primary care systems “serve as the patient’s first point of entry into the health care system and the continuing focal point for all needed health services”. To that end, they feature a set of primary care physicians (PCPs) who provide “primary care services to a defined population of patients”. These include “health promotion, disease prevention, health maintenance, counseling, patient education, diagnosis and treatment of acute and chronic illnesses”.

Demographic change challenges the functioning of primary care systems: Medical and technological progress paired with improved living conditions and reduced birth rates leads to an increased share of elderly citizens. In the United States, the percentage of individuals aged 65 and older is predicted to exceed 21 % of the total population by 2030 [65]. As populations age, their demand for primary care services tends to increase due to the prevalence of chronic illnesses, which disproportionately affect older adults [4, 46]. Simultaneously, primary care physicians are also aging; e.g., 34.1 % of all primary care physicians in Germany were 60 years or older by the end of 2017 [2] and thus about to retire. Moreover, fewer medical students are willing to practice primary care [46], let alone open a private primary care practice [35]. This reduces treatment capacities and exacerbates the risk for supply disruptions.

In the United States, the “confluence of a rising demand for primary care services and a decreasing supply of professionals providing these services” is considered a “crisis in primary care” [46]. In order to manage this crisis, existing systems have to adjust fundamentally [50]. Various new concepts and policies to maintain the standard of health care provision are discussed by the statutory health insurances, governments, and the Associations of Statutory Health Insurance Physicians [46, 56]. This discussion commonly distinguishes (i) microsystem improvements, which aim at enhancing a single server of the system and can be implemented at an individual level, and (ii) macrosystem reforms, which are fundamental, system-wide changes that must be implemented by policy makers [69]. Both types of system changes require validation and evaluation prior to their potentially costly implementation [50]. Naturally, this leads to the pressing question: How can we quantify the quality of primary care systems and the effects of changes?

The predominant solution to this problem is to assess the ratio between physicians and the population. In the United States or example, the Health Resources and Services Administration defines adequate health care supply based on profession- and region-specific population-to-provider ratios [16]. Similar ratio-based measures are applied in several European countries like Germany, Italy, and Spain [24, 45]. Such ratio-based assessments have several shortcomings: Even if they incorporate the local situation, ratios can only provide a very rough estimate. Furthermore, adjustment criteria are highly dependent on the definition of the underlying zones or geographic areas. They neglect factors such as the accessibility of practices and PCPs’ individual workloads. Finally, ratio-based assessments cannot account for new concepts such as telemedicine, mobile medical units, or centralized appointment scheduling.

**Fig. 1:**
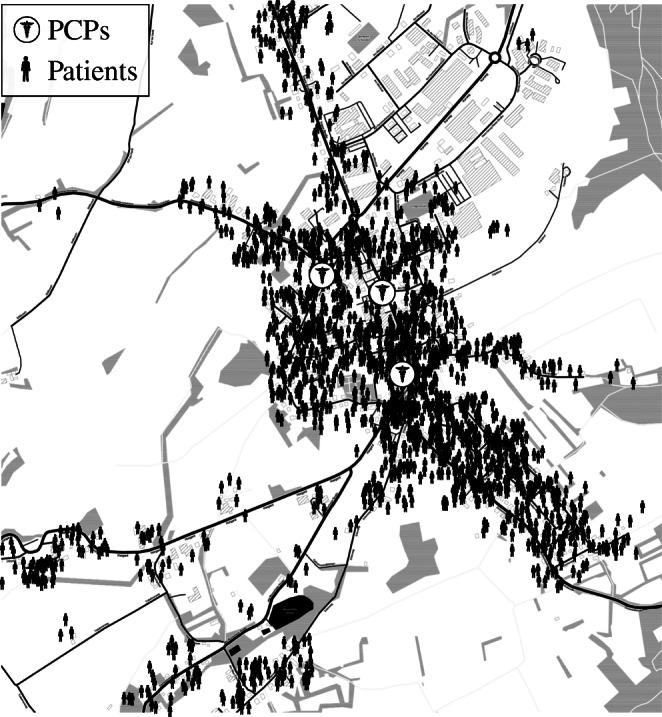
Geo-social system of patients and physicians. Note: Map tiles by Stamen Design, under CC BY 3.0. Data by OpenStreetMap, under ODbL

To overcome these limitations, this paper contributes the hybrid agent-based simulation tool SiM-Care (**Si** mulation **M** odel for Primary **Care**). SiM-Care represents patients and PCPs on an individual level as illustrated by Fig. 1. It models patients and primary care physicians via a geo-social system, in which patients decide whether and where to request an appointment and PCPs handle appointment requests, manage patient admission, and treat patients. By tracking the resulting interactions in SiM-Care, planners can identify dependencies of subproblems, evaluate new planning approaches, and quantify the effects of interventions on the basis of multiple key performance indicators. As such, SiM-Care can serve as a versatile decision support tool for primary care planning that is very generic, can be easily modified, and can be extended to meet individual needs. Based on empirical data from a German primary care system, we illustrate how to generate simulation scenarios and showcase SiM-Care through a case study. To the best of our knowledge, SiM-Care is the first simulation model that captures entire primary care systems with all physicians and patients as individual agents and allows for the simultaneous consideration of microsystem improvements as well as macrosystem reforms. The open source release of SiM-Care is currently in preparation.

The remainder of this paper is structured as follows. Section 2 discusses related work. We introduce SiM-Care on the basis of the ODD framework [26] in Section 3. Section 4 presents a case study based on real-world data to aid model validation and showcases the application of SiM-Care in health care planning. Section 5 summarizes the use and requirements of SiM-Care and further model applications before outlining future research.

## Related work

Decision support for health care planning is of increasing importance [29]. Related tools have to deal with the detail complexity that is inherent to the health care sector, finding it difficult to rely on the principle of “keep it simple, stupid” (KISS, [12]). Simulation modeling can deal with this complexity by “simulating the life histories of individuals and then estimating the population effect from the sum of the individual effects” [23]. As such, simulation models represent a powerful tool to inform policy makers: They can provide valuable insights into the dependencies within health care systems and allow for the prediction of the outcome of changes in strategy ahead of potentially costly and risky real-world interventions [23, 29].

Given these potentials, the use of computer simulation in health care delivery has significantly increased over the recent years [69]. The resulting body of literature is rich, as shown by several surveys of existing contributions. Examples include [15, 23], who review the use of simulation modeling for health care in general. Other surveys are mostly focused on particular simulation paradigm, e.g., system dynamics [11, 32], discrete event simulations [29, 36], agent-based modeling [7, 64], and hybrid simulations [13, 14]. Most recently, with general research attention being focused on the matter of pandemics in general and COVID-19 in particular, [20] point out opportunities for health care simulation modeling for pandemics beyond epidemiological modeling. The authors list a variety of decisions in emergency health care that simulation modeling, such as exemplified by SiM-Care, can support. Nevertheless, some sources remark that research on health care modeling continues to be under-reported [10] and highlight a “lack of real-world involvement in published simulation modelling” [15].

As background for the primary contribution of presenting a novel simulation system, we consider several examples of the computational study of primary care systems. Related references stem from a literature research featuring the keywords {simulation, decision support, system dynamics, discrete event, agent based model} + {primary care, health care}. Table 1 lists the resulting sources and differentiates the simulation paradigm, the modeling objective, and information on stakeholder involvement and maintenance. Accordingly, we broadly partition the considered models into two groups: those studying microsystem improvements and those investigating macrosystem reforms.

**Table 1:** Classification of related simulation models in primary care

Ref.	Method	Setting	Objective	Stakeholder Involvement	Maintenance
[18]	DES	Single primary care clinic	Eval. sequencing- and appointment rules	No information	No information, only management recommendations
[25]	DES	Single outpatient clinic	Testing a new scheduling approach	Stakeholder involvement through action research	No information, implemented recommendations
[59]	DES	Single primary care clinic	Eval. of appointment systems	No information	No information on maintenance, emphasize adaptability
[61]	DES	Single primary care clinic	Eval. effects of six factors on clinic’s performance	Management involvement in data collection	Researchers provided only recommendations, no system
[67]	DES	Single primary care clinic	Eval. implications of capacity allocations and appointment scheduling	Aimed to support stakeholders, no explicit involvement	No information on availability and maintenance
[69]	DES	Single pediatric clinic	Eval. effects of scheduling templates, staff ratios, room assignments	Analysis of exemplary clinic, no information on stakeholder involvement	No information
[31]	ABM	Entire health care system	Investigate paradox of primary care	Cooperation between academics and patients, caregivers, and clinicians	Model, software, and worksheets available for download and discussion
[47]	SD	Entire primary care sector	Eval. effects of system-wide policy changes	Group model building, development workshop	Model handed over to Regional Health Systems

Studies of microsystem improvements include [18, 25, 59, 61, 67, 69]. In contrast to SiM-Care, these models only feature a single primary care practice. Moreover, all of these models adopt a different approach to the representation of patients: While SiM-Care models a persistent patient population that is shared by all providers, the referenced models represent patients only as they arrive at the practice and disregard their evolution when they are discharged. As a result, such models do not account for the effects of individual microsystem improvements on the entire system.

Other references, such as [31, 47], investigate macrosystem reforms and feature entire primary care systems. Still, the agent-based model [31] differs from SiM-Care in its objective: It investigates the external effects of treatments in primary care on the entire health care system, whereas SiM-Care focuses on the processes within primary care systems. Hence, [31] does not model internal processes, such as appointment scheduling. Model [47] implements the system dynamics paradigm and thus focuses on a higher level system representation than SiM-Care. While system dynamics models do not consider the level of micro-detail offered by agent-based simulations, they require less computational effort to run simulation experiments. In addition, they may provide a more concise model that is easier to communicate to stakeholders. This motivates us to specifically consider aspects of model validation in the case study.

To the best of our knowledge, no existing simulation model allows the simultaneous consideration of microsystem improvements and macrosystem reforms in primary care systems that SiM-Care provides.

## Simulation model

Creating a simulation model means both formalizing what the model includes and deciding what to leave out [64]. Therefore, this section first discusses the process of creating the model and the involvement of stakeholders before listing the resulting model assumptions and limitations. Subsequently, we formally describe all modeled components and relationships.

SiM-Care is designed to meet the requirements of various stakeholders. *Researchers* access the model to evaluate outcomes from prescriptive planning approaches based on mathematical modeling. The modeling team regularly consulted with health care practitioners including *primary care physicians*, *health insurance representatives*, as well as *representatives from industry associations* and *administrative authorities*. Generally, we find that explaining the simulation model through the agent-based paradigm and presenting results from related studies allows for in-depth discussions, where the simulation provides a helpful tool for illustration.

At an early modeling stage, it became evident that the model would never be able to mirror all intricacies of a primary care system. Therefore, development focused on the idea of “modeling the problem, not the system”, as recommended by [49]. Here, the primary problem is evaluating the macro-level effects from combining of health care supply in the form of a population of physicians versus a demand in the form of a population of patients.

Thereby, we model the trade-offs between the objectives pursued by three stakeholder groups: patients, PCPs, and policy makers. SiM-Care assumes that PCPs strive to efficiently utilize their time, whereas patients strive for a quick response to their health concerns. Thereby, the model illustrates the trade-off between efficiency and patient-centered care. Policy objectives can range from minimizing the cost of health care to maximizing the degree of patient-centered care. Policy makers are not represented by agents within SiM-Care. Instead, policy decisions set relevant model parameters such as the number of physicians in the system and treatment standards. To model interactions on a micro-level, SiM-Care thus features two populations of agents: potential patients $\mathcal {P}$ and primary care physicians $\mathcal {G}$.

**Fig. 2:**
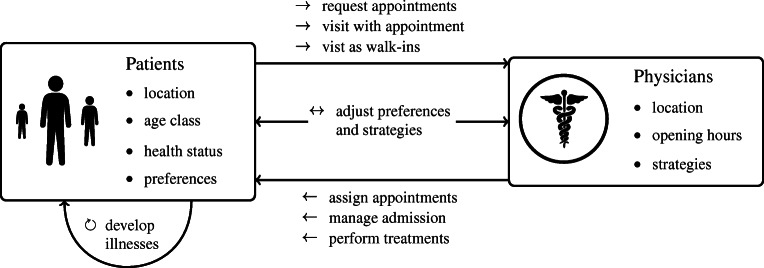
Concept of SiM-Care showing both types of agents with their main attributes as well as interactions between agents

Every patient $\rho \in \mathcal {P}$ resides at a specific location, belongs to a certain age group and has an individual health status and treatment preferences; compare Fig. 2. Patients develop acute illnesses that depend on their age and health status and require treatment. Additionally, patients may suffer from long term chronic illnesses, which need to be monitored by a physician. To receive medical attention, patients either schedule an appointment or visit a PCP’s practice without prior notice. Patients’ decisions depend on their individual preferences and health status. These factors determine the choice of physician, the type of the visit (walk-in/ appointment), and the time of the visit.

All PCPs $\phi \in \mathcal {G}$ practice at a certain location and have weekly opening hours; see Fig. 2. Moreover, every physician $\phi \in \mathcal {G}$ follows individual strategies that govern how they manage appointments, admit patients, and perform treatments. As patients and physicians interact, they influence each other and adjust their preferences and strategies.

In the following, we list – to the best of our knowledge – the underlying assumptions and the limitations that may restrict the application of SiM-Care.

SiM-Care focuses on the adult population and neither models pediatric care nor gender differences. While we do differentiate patients by health status, age, and illnesses, we assume that all patients implement the same strategies when arranging appointments or becoming walk-ins. Furthermore, the model assumes that all patients attend their appointments, i.e., there are no no-shows patients.

As it stands, the model does not consider cross-effects between illnesses that may occur, e.g., when a chronic illness worsens the progression of an acute illness. As there is no model of direct patient interaction, SiM-Care does not include an explicit infection model, i.e., the probability of a patient developing an acute illness is independent of their interaction with other patients and physicians. While patients who suffer from illnesses seek treatment, the duration of an illness is not directly affected by treatments.

On the provider side, we do not model a relationship between primary care systems and specialists or hospitals. Physicians do not differentiate patients according to their insurance policy. The physicians’ service times do not depend on the patients’ number or types of illnesses and physicians do not offer home visits. We assume that PCPs are never late or absent and the model includes neither seasonality nor holidays. Finally, we assume independence of surrounding municipalities, such that the modeled primary care system is a closed system.

In the remainder of this section, we provide the model’s formal description based on the ODD framework described by Grimm et al. [26]. For the sake of brevity, some of the very technical modeling details are only presented in Appendix A.

### Simulation environment

SiM-Care’s environment entails the geographical and temporal structure as well as policy effects. Within the model, locations $\ell \in {\mathscr{L}} := [-90, 90] \times [-180,180]$ are represented using the geographic coordinates latitude and longitude.

The modeled time period is considered as a continuum structured by points in time and durations. For any time object $t=(\delta , \eta ) \in \mathcal {T} := \mathbb {N} \times [0,1) $, $\delta \in \mathbb {N}$ indicates the day and $\eta \in [0,1) =: {\mathscr{H}}$ specifies the time as an increment of day known as *decimal time*. That is, we use the same encoding for points in time and durations as context uniquely defines which of the former a time object refers to. For example, $(38,0.55) \in \mathcal {T}$ corresponds to day 38 and 24 ⋅ 60 ⋅ 0.55 = 792 minutes, i.e., 1:12 p.m. as a point in time or, analogously, to a duration of 38 days, 13 hours, and 12 minutes. To ease notation, we associate every point in time and duration $(\delta , \eta ) \in \mathcal {T}$ with the non-negative value $\delta +\eta \in \mathbb {R}_{\geq 0}$.

In addition to the continuous representation of time, we structure each day into a morning and an afternoon session as it is common practice in primary care [40]. Each *session* $\lambda =(\delta ,\gamma ) \in {\Lambda } := \mathbb {N} \times \{0,1\}$ is uniquely defined by a day $\delta \in \mathbb {N}$ and a binary indicator *γ* ∈{0,1} that defines whether it is the morning (*γ* = 0) or the afternoon (*γ* = 1) session. Sessions reoccur on a weekly basis which yields an equivalence relation $\sim $ on the set of sessions Λ via

$$(\delta_{1}, \gamma_{1}) \sim (\delta_{2}, \gamma_{2}) :\Leftrightarrow \delta_{1}\equiv \delta_{2} \text{mod} 7 \land \gamma_{1}= \gamma_{2}. $$

The resulting equivalence class for a session *λ* ∈Λ defined as $[\lambda ] := \{ \lambda ^{\prime } \in {\Lambda } : \lambda ^{\prime } \sim \lambda \}$ contains all sessions sharing the same day of the week and time of the day, e.g., all Thursday afternoon sessions. Thus, we model and distinguish 14 sessions each week, i.e., Monday to Sunday with a respective morning and afternoon session that we associate with the set of all equivalence classes ${\Lambda }{/}{\sim } := \{[\lambda ] : \lambda \in {\Lambda }\}$. Particularly, this allows for a distinction between sessions on weekdays and weekends.

### Entities and state variables

Patients $\rho \in \mathcal {P}$ and PCPs $\phi \in \mathcal {G}$ are the active entities in the simulation. Their interaction is motivated by patients’ suffering from illnesses and therefore seeking treatment with PCPs via appointments or walk-in visits.

Going from simple to more elaborated, we begin by describing the self-containing entities of SiM-Care and end with the description of the agents representing patients and physicians.

#### Objectives

When patients suffer from an acute illness, they want to be treated as soon as possible, ideally by their preferred physician. For the treatment of chronic illnesses and the follow-up care of acute illnesses, patients prefer treatment by the same physician through appointments in regular intervals. Physicians, on the other hand, aim at efficiently utilizing their available time while minimizing overtime. Thus, patients’ and physicians’ objectives conflict, as it is ineffective for physicians to fully comply with patient demands: To ensure that all short-notice appointment requests can be accommodated, PCPs would have to withhold too much treatment time. Providing follow-up appointments in strict intervals would prevent PCPs from reacting to demand fluctuations.

Policy makers follow a multitude of conflicting objectives. On the one hand, they need to ensure a certain minimum standard in health care quality to guarantee patients are treated when necessary. On the other hand, they cannot afford to subsidize an excessive number of physicians. Thus, policy makers necessarily aim at a trade-off.

SiM-Care represents policy decisions through their resulting parameter values, e.g., the number of physicians and their distribution.

#### Illnesses and families of illnesses

Illnesses are health concerns that cause discomfort to patients and require treatment. They belong to a certain illness family (e.g. cold or heartburn), have a certain seriousness (e.g. mild or severe), persist over a certain period of time, and require initial treatment within an acceptable time frame as well as subsequent follow-up visits in regular time intervals. SiM-Care formalizes illnesses as tuples $i=(s_{i}, f_{i},d_{i}, \omega _{i}, \nu _{i}) \in {\mathscr{I}}$ with attributes as shown in Table 2. Thereby, *s*_*i*_ ∈ [0,1] defines the seriousness of the illness, $f_{i} \in \mathcal {F}$ defines the illness family, and $d_{i} \in \mathcal {T}$ defines the duration. The parameter $\omega _{i} \in \mathcal {T}$ defines the willingness to wait, which is the patient’s maximum accepted waiting time for the initial treatment of this illness. The parameter $\nu _{i} \in \mathcal {T}$ defines the follow-up interval, which specifies the frequency of the required aftercare that follows the initial treatment of this illness. For some illnesses $i\in {\mathscr{I}}$, the characteristics duration and follow-up interval do not apply. This is indicated by setting *d*_*i*_ = *∅* and *ν*_*i*_ = *∅*.

**Table 2:** Attributes of illnesses $i\in {\mathscr{I}}$

Attribute	Type	Unit
seriousness	*s*_*i*_ ∈ [0, 1]	
illness family	$f_{i} \in \mathcal {F}$	
duration	$d_{i}\in \mathcal {T} $	[days]
willingness to wait	$\omega _{i} \in \mathcal {T}$	[days]
follow-up interval	$\nu _{i}\in \mathcal {T} $	[days]

While emerging illnesses vary in their manifestation, families of illnesses define the common constant traits of all illnesses belonging to the same family. In our model, the common constant traits of all illnesses $i\in {\mathscr{I}}$ with seriousness *s*_*i*_ ∈ [0,1] belonging to family $f_{i}\in \mathcal {F}$ are the expected duration $D_{f_{i}}(s_{i}) \in \mathcal {T}$, the expected willingness to wait $W_{f_{i}}(s_{i}) \in \mathcal {T}$, and the follow-up interval $N_{f_{i}}(s_{i}) \in \mathcal {T}$. The expected duration $D_{f_{i}}(s_{i})$ and expected willingness to wait $W_{f_{i}}(s_{i})$ serve as means for the distributions, from which we sample each stochastic duration *d*_*i*_ and stochastic willingness to wait *ω*_*i*_. Thus for all emerged illnesses $i\in {\mathscr{I}}$, it generally holds that $d_{i} \neq D_{f_{i}}(s_{i})$ and $\omega _{i} \neq W_{f_{i}}(s_{i})$. Only the follow-up interval of emerged illnesses $i\in {\mathscr{I}}$ derives from the illness family in a deterministic way, i.e., $\nu _{i}=N_{f_{i}}(s_{i})$.

In order to define the common traits of illnesses, families of illnesses $f \in \mathcal {F}$ are specified by the three linear functions shown in Table 3. As above, we indicate the inapplicability of the characteristics duration or follow-up interval to families of illnesses by setting *D*_*f*_ = *∅* and *N*_*f*_ = *∅*, respectively.

**Table 3:** Attributes of families of illnesses $f \in \mathcal {F}$

Attribute	Type
linear function for expected duration	$D_{f}\colon [0,1] \to \mathcal {T}$
linear function for expected willingness	$W_{f}\colon [0,1] \to \mathcal {T}$
linear function for follow-up interval	$N_{f} \colon [0,1] \to \mathcal {T}$
chronic attribute	*κ*_*f*_ ∈{0, 1}

To illustrate this concept, consider the family “common cold” defined by the functions *D*_*f*_(*s*) = 10*s* + 3, *W*_*f*_(*s*) = − 3*s* + 3, and *N*_*f*_(*s*) = − 2*s* + 7. When a patient develops a mild case of “common cold” (*s*_*i*_ = 0.2), the illness family “common cold” defines the expected duration, expected willingness to wait, and follow-up interval of the mild cold as *D*_*f*_(*s*_*i*_) = 5 days, *W*_*f*_(*s*_*i*_) = 2.4 days, and *N*_*f*_(*s*_*i*_) = 6.6 days. The actual values of the specific cold are stochastic and vary around their expected counterparts, e.g., *d*_*i*_ = 5.5 days and *ω*_*i*_ = 2.7 days. The follow-up interval is deterministic and derives from the illness family via $\nu _{i}=N_{f_{i}}(s_{i})=6.6$ days. Note, that the particular mild cold in this example will thus not require a follow-up visit, as its duration is shorter than the follow-up interval, i.e., *d*_*i*_ < *ν*_*i*_.

To model chronic health concerns that persist over an extended period of time, such as diabetes, a chronic attribute *κ*_*f*_ ∈{0,1} identifies families of chronic illnesses. Thereby, *κ*_*f*_ partitions $\mathcal {F}$ into an acute set $\mathcal {F}^{\text {act}}:= \{f\in \mathcal {F}: \kappa _{f}=0\}$ and a chronic set $\mathcal {F}^{\text {chro}}:= \{f\in \mathcal {F}: \kappa _{f}=1\}$. This directly induces a partition of the set of illnesses ${\mathscr{I}}$ into acute and chronic illnesses ${\mathscr{I}}^{\text {act}}$ and ${\mathscr{I}}^{\text {chro}}$.

Acute illnesses $i \in {\mathscr{I}}^{\text {act}}$ develop and subside over time and patients can simultaneously suffer from an arbitrary number of acute illnesses $\mathcal {I}^{\text {act}} \subseteq {\mathscr{I}}^{\text {act}}$. Chronic illnesses ${\varsigma }\in {\mathscr{I}}^{\text {chro}}$ are static in SiM-Care – they neither develop nor heal. Instead, each patient $\rho \in \mathcal {P}$ suffers from at most one chronic illness throughout the modeled time period. To distinguish patients suffering from a chronic illness, we refer to them as *chronic patients*.

Ideally, attributes of families of illnesses should be estimated from empirical data. For example, follow-up intervals can be estimated from collections of individual treatment histories that indicate the times at which patients were treated for a particular diagnosis. In case empirical data is not available, estimates can be based on expert knowledge or official treatment guidance such as disease management programs [6]. Moreover, if one does not aim to make detailed predictions for a specific primary care system, it is possible to infer attributes or model entirely artificial health concerns as long as they are sufficiently validated in a baseline analysis. This last and most basic approach is adopted in the case study presented in Section 4.

#### Appointments

Appointments specify the point in time when the treatment of a specific patient is scheduled to take place. To that end, appointments $b\in {\mathscr{B}}$ are defined by the time of the appointment $t_{b} \in \mathcal {T}$, the attending primary care physician $\phi _{b} \in \mathcal {G}$, and the patient $\rho _{b} \in \mathcal {P}$ receiving treatment. At any point in time, non-chronic patients can have at most one scheduled appointment $b^{\text {act}}\in {\mathscr{B}}$, called the *acute* appointment. Acute appointments are intended for the initial treatment of acute illnesses, the follow-up treatment of acute illnesses, or both. Chronic patients may additionally have a *regular* appointment $b^{\text {reg}}\in {\mathscr{B}}$ to treat their chronic illness. While chronic illnesses are only treated during regular appointments, all acute illnesses $\mathcal {I}^{\text {act}}$ are treated during every appointment.

#### Age classes

Age classes define common characteristics of patients. For patients of age class $a\in \mathcal {A}$, these characteristics are deviations from the expected illness duration ${\Delta }^{d}_{a} > 0$ and from the expected willingness to wait ${\Delta }^{\omega }_{a} \geq 0$, the probability to cancel an appointment after full recovery *p*_*a*_ ∈ [0,1], and the expected number of annual acute illnesses defined through the linear function $I_{a}\colon [0,1] \to \mathbb {R}_{\geq 0}$; see Table 4. The deviation from the expected illness duration ${\Delta }^{d}_{a}$ is a multiplicative factor that determines whether the expected illness duration $D_{f_{i}}(s_{i})\in \mathcal {T}$ extends (${\Delta }^{d}_{a}>1$) or shortens (${\Delta }^{d}_{a}<1$) for patients of age class $a\in \mathcal {A}$; analogously for ${\Delta }^{\omega }_{a}$. The linear function $I_{a}\colon [0,1] \to \mathbb {R}_{\geq 0}$ defines the expected number of annual acute illnesses $I_{a}(c)\in \mathbb {R}_{\geq 0}$ for patients in age class $a \in \mathcal {A}$ which depends on the patient’s health condition *c* ∈ [0,1] which can range from perfectly healthy (*c* = 0) to extremely delicate (*c* = 1).

**Table 4:** Attributes of age classes $a \in \mathcal {A}$

Attribute	Type
linear function expected annual acute illnesses	$I_{a}\colon [0,1] \to \mathbb {R}_{\geq 0}$
deviation from expected illness duration	${\Delta }^{d}_{a}>0$
deviation from expected willingness to wait	${\Delta }^{\omega }_{a} \geq 0$
probability to cancel appointments	*p*_*a*_ ∈ [0, 1]

#### Age-class-illness distribution

The age-class-illness distribution $\pi ^{\text {act}} \colon \mathcal {A} \times \mathcal {F}^{\text {act}} \to [0,1]$ builds the connection between the set of age classes $\mathcal {A}$ and the set of acute families of illnesses $\mathcal {F}^{\text {act}}$. To that end, *π*^act^ defines the expected distribution of acute illness families per age class, i.e., among all developed acute illnesses by patients of age class $a \in \mathcal {A}$, a fraction *π*^act^(*a*, *f*_*i*_) ∈ [0,1] is expected to belong to illness family $f_{i}\in \mathcal {F}^{\text {act}}$.

#### Patients

Patients are the driving force of the simulation, as their health concerns trigger most events. All non-chronic patients $\rho \in \mathcal {P}$ are characterized by their geographical location $\ell \in {\mathscr{L}}$, health condition *c* ∈ [0,1], acute illnesses $\mathcal {I}^{\text {act}} \subseteq {\mathscr{I}}^{\text {act}}$, age class $a\in \mathcal {A}$, acute appointment $b^{\text {act}} \in {\mathscr{B}}$, and preferences. While the location, health condition, and age class of each patient remain constant throughout a simulation experiment, a patient’s acute illnesses, acute appointment and preferences can change over time. Chronic patients are additionally characterized by a constant chronic illness ${\varsigma } \in {\mathscr{I}}^{\text {chro}}$ and a variable regular appointment $b^{\text {reg}}\in {\mathscr{B}}$. Table 5 summarizes the attributes shared by all patients as well as the attributes specific to chronic patients.

**Table 5:** Attributes of (chronic) patients $\rho \in \mathcal {P}$

Attribute	Domain	Type
location	$\ell \in {\mathscr{L}}$	constant
health condition	*c* ∈ [0, 1]	constant
age class	$a \in \mathcal {A}$	constant
acute illnesses	$\mathcal {I}^{\text {act}} \subseteq {\mathscr{I}}^{\text {act}}$	variable
emergency flag	*ε* ∈{0, 1}	variable
acute appointment	$b^{\text {act}} \in {\mathscr{B}}$	variable
considered PCPs	$\mathcal {G}^{\text {con}} \subseteq \mathcal {G}$	constant
availabilities	$\alpha \colon {\Lambda }{/}{\sim } \to \{0,1\}$	constant
appointment ratings	$r_{\rho }^{\text {app}} (\phi ) \geq 0, \forall \phi {\in } \mathcal {G}^{\text {con}}$	variable
walk-in ratings	$r_{\rho }^{\text {walk}} (\phi , [\lambda ]) \geq 0,$	variable
	$\forall \phi {\in } \mathcal {G}^{\text {con}}, \forall [\lambda ]{\in }{\Lambda }{/}{\sim } $	
chronic illness	$\mathcal {I}^{\text {chro}}= \{{\varsigma }\} \subseteq {\mathscr{I}}^{\text {chro}}$	constant
regular appointment	$b^{\text {reg}} \in {\mathscr{B}}$	variable
family physician	$\phi ^{\text {fam}} \in \mathcal {G}^{\text {con}}$	variable

Patients’ preferences determine when, where, and how they pursue treatment. Specifically, each patient considers a set of PCPs $\mathcal {G}^{\text {con}} \subseteq \mathcal {G}$ and never seeks treatment with PCPs outside the consideration set. Since continuity in the treatment of chronic illnesses is particularly important, chronic patients arrange all regular appointments $b^{\text {reg}}\in {\mathscr{B}}$ with a distinguished family physician $\phi ^{\text {fam}} \in \mathcal {G}^{\text {con}}$. While every patients’ consideration set $\mathcal {G}^{\text {con}}$ remains constant throughout the modeled time period, patients reevaluate and vary their family physician. Naturally, patients have personal schedules and cannot attend all weekly sessions. Thus, the model assumes that each patient has a constant set of weekly-reoccurring session availabilities given by $\alpha \colon {\Lambda }{/}{\sim } \to \{0,1\}$, where 0 encodes unavailability. Finally, patients maintain individual appointment ratings $r_{\rho }^{\text {app}} (\phi ) \geq 0$ as well as session-specific walk-in ratings $r_{\rho }^{\text {walk}} (\phi , [\lambda ]) \geq 0$ for every weekly session $[\lambda ] \in {\Lambda }{/}{\sim }$ and every considered physician $\phi \in \mathcal {G}^{\text {con}}$.

Via internal ratings, patients track their satisfaction with a physician’s services. Whenever a patient seeks consultation, the choice of physician is determined by their current ratings. Ratings incorporate patients’ sense of geographic distance, matching of opening hours with availabilities, and previous positive and negative experiences. As patients adjust their ratings over time, they adjust their choice of PCP. If a physician is unable to meet an appointment request, incurs excessive waiting time, or rejects patients due to capacity overruns, patients reduce their rating. Positive experiences such as successful appointment arrangements or short waiting times increase ratings. Note that ratings are only internal valuations and not communicated to other patients or physicians.

When patients begin to suffer from a new illness, they always seek treatment. To that end, patients first request an appointment from the set of considered PCPs, $\mathcal {G}^{\text {con}}$. Patients make up to two appointment requests in order of the appointment rating $r_{\rho }^{\text {app}} (\phi ) \geq 0$. If both requested PCPs fail to offer a feasible appointment within the patient’s willingness to wait, patients forgo an appointment and visit a PCP as a walk-in. They select the PCP for the walk-in visit based on the walk-in rating $r_{\rho }^{\text {walk}} (\phi , [\lambda ])$ of the targeted session *λ* ∈Λ.

Upon arrival, a PCP may reject patients due to, e.g., capacity overloads. Following a rejection, patients update their rating of this PCP and attempt a new walk-in visit at the then-highest-rated PCP. Rejected patients are flagged as emergencies (*ε* = 1) for as long as they unsuccessfully continue to seek treatment. PCPs may include the emergency state in their decision making.

Until an illness $i\in \mathcal {I}^{\text {act}}$ subsides, patients continuously arrange follow-up appointments with the attending physician in the follow-up interval $\nu _{i}\in \mathcal {T}$. Analogously, chronic patients continuously arrange regular appointments with their family physician $\phi ^{\text {fam}} \in \mathcal {G}^{\text {con}}$ in the follow-up interval $\nu _{{\varsigma }}\in \mathcal {T}$ of their unique chronic illness ${\varsigma }\in \mathcal {I}^{\text {chro}}$.

#### Primary care physicians

PCPs’ practices feature an uncapacitated waiting room. The model characterizes physicians $\phi \in \mathcal {G}$ by their geographic location $\ell \in {\mathscr{L}}$, opening hours, as well as an individual set of strategies to schedule appointments, manage patient admission, and organize treatments. Table 6 summarizes the attributes of PCPs.

**Table 6:** Attributes of PCPs $\phi \in \mathcal {G}$

Attribute	Type
location	$\ell \in {\mathscr{L}}$
opening hours	$o\colon {\Lambda }{/}{\sim } \to {\mathscr{H}} \times {\mathscr{H}}$
appointment scheduling strategy	$S\in \mathcal {S}^{\text {app}}$
admission strategy	$S \in \mathcal {S}^{\text {adm}}$
treatment strategy	$S \in \mathcal {S}^{\text {tmt}}$

**Fig. 3:**
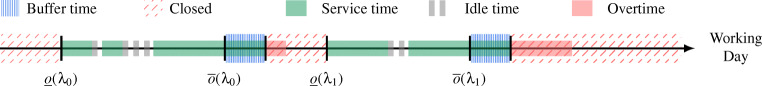
Schematic representation of a PCP’s morning (*λ*_0_) and afternoon (*λ*_1_) session visualizing service-, idle- and overtime

SiM-Care assumes that all physicians operate in clinical sessions. Opening hours for these sessions are weekly-reccurring and therefore defined over the session of the week via $o\colon {\Lambda }{/}{\sim } \to {\mathscr{H}} \times {\mathscr{H}}$ where ${\mathscr{H}}$ denotes the set of decimal times; cf. Section 3.1. Opening hours specify for each session *λ* ∈Λ the time window $o([\lambda ]):= [\underline {o}([\lambda ]), \overline {o}([\lambda ])]$ during which patients are admitted. The beginning of session *λ* = (*δ*, *γ*) ∈Λ is defined as $\underline {o}(\lambda ):= (\delta ,\underline {o}([\lambda ]) \in \mathcal {T}$, the session’s end as $\overline {o}(\lambda ):= (\delta ,\overline {o}([\lambda ]) \in \mathcal {T}$. To encode that a PCP is closed, we set *o*([*λ*]) = *∅*. Physicians use the first hour after the end of each session as time buffer to compensate for possible delays and walk-in patients. Figure 3 visualizes a PCP’s working day.

PCPs implement a set of *strategies* to schedule appointments, decide on patient admissions, and organize the treatment of patients. These strategies are interchangeable model components that are defined via interfaces. They govern the physicians’ interactions with patients in terms of sensing, predicting, adapting, and learning. In the following, we summarize the main functionality of each strategy. All technicalities and the exemplary strategies that are used as part of our case study can be found in Appendix A.1.

The PCP’s *appointment scheduling strategy* $S\in \mathcal {S}^{\text {app}}$ defines how they allocate consultation time to appointment slots and how the resulting slots are assigned to requesting patients.

The PCP’s *treatment strategy* $S\in \mathcal {S}^{\text {tmt}}$ defines the order of treatment based on patients’ waiting times. To account for the observation that physicians consciously or unconsciously adjust service times depending on demand [28], treatment policies define when and how physicians adjust their consultation speed and thereby service times.

The PCP’s *admission strategy* $S\in \mathcal {S}^{\text {adm}}$ determines whether they admit an arriving patient based on the current workload. SiM-Care requires PCPs to treat all admitted patients. Thus, when physicians underestimate their workload, they may have to work overtime. On the other hand, physicians that overestimate their workload fail to fully utilize their available time. At the end of a session’s buffer, physicians may learn by reevaluating their predictions and adapting their admission policy.

### Process overview and scheduling

SiM-Care models the progression of time via the discrete event paradigm. Thus time passes between discrete events, at which the system state is updated. The model stores events of the form (*t*, *e*) in a sequential queue $\mathcal {Q}$ where $t\in \mathcal {T}$ is the point in time an event of type $e\in \mathcal {E}$ occurs. By construction, the event queue $\mathcal {Q}$ never runs empty.

**Fig. 4:**
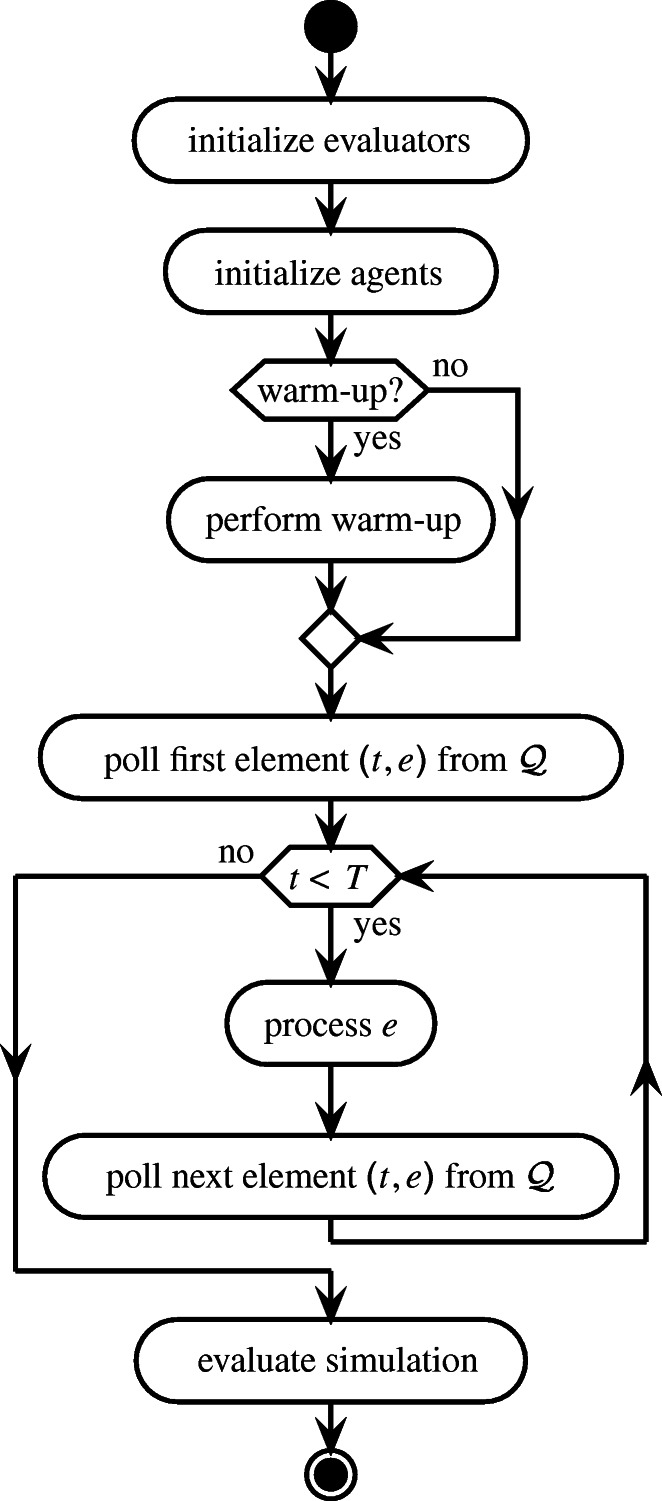
Structure of simulation run with time horizon *T*

Every simulation run follows the structure depicted in Fig. 4, chronologically processing the events from $\mathcal {Q}$ up to a specified point in time $T \in \mathcal {T}$. In this, the specific process depends on the event type $e\in \mathcal {E}$. Appendix A.2 details the modeled events and their processing.

### Modeling variability

SiM-Care relies on stochastic values to approximate real-world variability and control the frequency of events. This applies to aspects of illnesses as well as to patient arrivals, appointment cancellations and service times. In consequence, every simulation experiment includes multiple stochastic repetitions of the modeled time period, termed *simulation runs*. When examining simulation output, we account for the resulting variability through confidence intervals.

Table 7, lists all probabilistic model aspects. Appendix A.3 details the parameterization of the distributions underlying the random values.

**Table 7:** Probabilistic model aspects

Aspect	Distribution
frequency of acute illnesses	exponential distribution
type of acute illnesses	age-class-illness distribution
seriousness of acute illnesses	triangular distribution
duration of acute illnesses	log-normal distribution
patients’ willingness to wait	Weibull distribution
patient punctuality	normal distribution
walk-in arrivals	beta distribution
service time	log-normal distribution
appointment cancellations	binomial distribution

### Emergence and observation

SiM-Care tracks multiple key performance indicators to illustrate the trade-offs between the stakeholders’ objectives. These indicators emerge from agent interactions based on patients’ evolving preferences and physicians’ evolving strategies. Appendix A.4 provides formal definitions of all evaluated key performance indicators. In the following we summarize them briefly.

From the patients’ point of view, key performance indicators include access time, access distance, and waiting time. To evaluate the patients’ indicators, SiM-Care keeps track of the total access time of arranging acute and regular appointments, the total number of arranged acute and regular appointments, the total number of attended appointments, the total number of walk-in patients, the total access distance of patients, and the total waiting time for both patients with appointment and walk-ins.

From the physicians’ point of view, key performance indicators include the utilization, overtime, number of treatments, and number of rejected patients with and without appointment. To evaluate the physician’s indicators, SiM-Care collects on physician level the total service time spent treating patients, the total number of performed treatments, the total overtime, and the total number of rejected patients with and without appointment. The total available working time per PCP required to compute the utilization, can be derived from the opening hours *o* and the modeled time horizon *T*.

### Input, initialization, and warm-up

SiM-Care codes many values as flexible parameters. Setting up a simulation experiment requires specifying the parameter values. Each simulation scenario represents a particular setting, in which a specific set of patients interacts with a specific set of physicians under specific circumstances.

As part of every simulation scenario, the modeler specifies the families of illness $\mathcal {F}$, the age classes $\mathcal {A}$, the age-class-illness distribution *π*^act^, and the set of physicians $\mathcal {G}$ with all their attributes. The set of patients $\mathcal {P}$ is only partially defined through the simulation scenario: Each scenario specifies every patient’s location $\ell \in {\mathscr{L}}$, health condition *c* ∈ [0,1], age class $a\in \mathcal {A}$, availabilities $\alpha \colon {\Lambda }{/}{\sim } \to \{0,1\}$, and, for chronic patients, a chronic illness ${\varsigma }\in {\mathscr{I}}^{\text {chro}}$. The remaining attributes of patients are derived as described in Appendix A.5.

To initialize a simulation experiment, modelers can broadly choose one of two approaches: empty and interim initialization. An empty system state is inherently unrealistic, as it sets all parameters that are subject to simulation dynamics to zero. An ideal interim initialization would mean that there is no period when the system state does not align with the real-world observations. However, this creates additional challenges for validation. For a structurally valid simulation, a valid interim state should automatically emerge from an empty state initialization after a warm-up period. Thus, we rely on an empty-state initialization and include a warm-up period, where the simulation state does not align with any plausible real-world state. The duration of the warm-up and the length of the modeled time horizon are both variable per experiment.

### Submodels

We consider various aspects of SiM-Care that rely on an internal logic as submodels. One of the most basic submodels describes the logic of distances and travel times. More complex examples include the logic underlying patients’ behavior when requesting appointments and visiting practices as walk-ins. Appendix A.6 provides the specifics of all submodels.

### Structural validation and verification

We carried out validation and verification for SiM-Care according to the best practices documented in the literature [42, 57]. To ensure a correct model implementation (verification), we followed established programming practices. We used object oriented programming to write modular code, implementing SiM-Care in Java 8. All random distributions rely on the Apache Common Math library [48]. We verified each module individually through unit testing. Assertions ensure that variables remain within their specifications at runtime. To detect undesired model behavior, SiM-Care can trace the entire simulation process. Traces are specialized logs that contain all information about the model’s execution. In SiM-Care, traces are textual and comprehensible to modelers. They can track agents throughout the model and contain all information that would be required for an animated visualization. By analyzing traces and input output relationships, we performed dynamics tests for multiple simulation scenarios of various sizes with different system setups.

To ensure that the conceptional model serves as an adequate representation of real primary care systems (validation), we took several measures. With regard to face validity, we presented the conceptual model to physicians and decision makers from health insurers as well as public authorities. Furthermore, SiM-Care builds on data from the literature as well as empirical data collected on-site. Moreover, we visited a primary care practice and interviewed staff to capture and understand the daily processes and routines of PCPs. For the specific scenarios featured in the case study, we validated the simulation output with available empirical data. Details on this historical validation can be found in the baseline analysis of the following case study.

## Case study

To demonstrate the potential use of SiM-Care, we present a case study evaluating the effects of changes in the population of a primary care system. Specifically, we create a baseline scenario representing a real-world primary care system and investigate two possible changes from the status quo. On the one hand, we let the number of PCPs decline as a result of a decreasing interest in opening a primary care practice in rural areas [35]. On the other hand, we let an aging population cause a shift in the quality and intensity of illnesses and the resulting health care requirements. By considering both changes individually and in combination, we create three “what-if” scenarios that we compare to the baseline scenario.

Each scenario models a time period of one year preceded by warm-up period. As SiM-Care relies on stochastic values, every simulation experiment includes 20 independent runs. While 20 repetitions are generally at the low end of the suggested number of runs, the resulting confidence intervals are sufficiently small to assess the qualitative effects of changes between scenarios.

### Baseline scenario

The real-world primary care system that serves as the template for our study comprises three predominantly rural municipalities in western Germany (Roetgen, Simmerath, and Monschau) with a total population of approximately 36000 inhabitants and 20 primary care physicians. For the considered primary care system, empirical data concerning the physicians’ distribution and opening hours was provided by the responsible department of public health or obtained from the responsible association of statutory health insurance physicians [37]. The distribution of patients and their demographic composition is available from the national census [33] and official population projections by the federal state [34]. The distribution of illnesses and their characteristics can be estimated from publications of health insurances and federal government agencies [27, 53]. All unavailable data was either empirically collected in a primary care practice or, where this was not possible, inferred. For the sake of clarity, Table 8 summarizes our basis for the selection of each input parameter.

**Table 8:** Basis for the selection of input parameters

Attribute	Basis (Source)
PCPs	
location	empirical (dept. public health)
opening hours	empirical ([37])
strategies	literature ([17, 39, 40])
Patients	
location	empirical ([33])
age class	empirical ([33])
health condition	inferred
Age classes	
exp. annual acute illnesses	inferred
dev. illness duration	inferred
dev. willingness to wait	inferred
availabilities	inferred
appointment cancellation	inferred
chronic patients	empirical ([53])
Families of Illnesses	
characteristics	inferred
age-class-illness dist.	empirical ([27])

In the following, we discuss how the available empirical data translates into a simulation scenario. To that end, we detail the input parameter choices, i.e., the modeled physicians, patients, age classes, families of illnesses, and age-class-illness distributions.

#### Primary care physicians

**Fig. 5:**
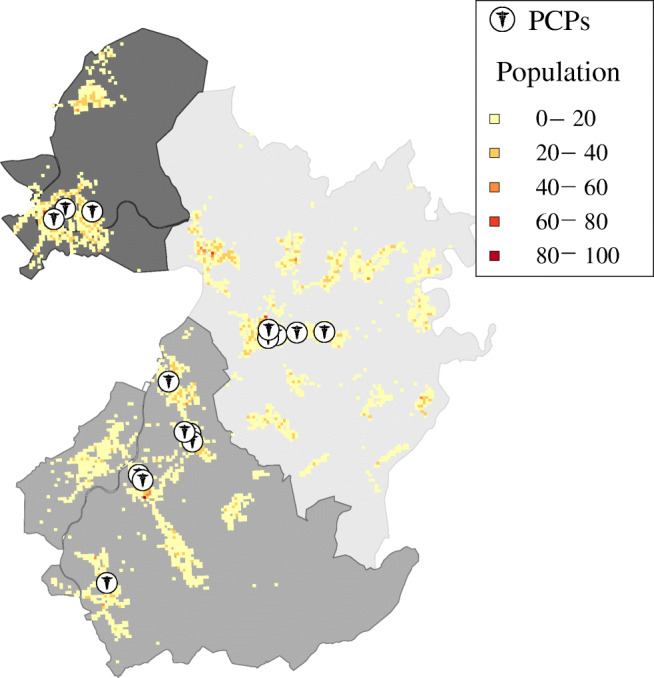
Locations of PCPs with health insurance accreditation and population cells reported by the 2011 census [33]

According to data provided by the Aachen department of public health in 2017, there are 20 primary care physicians with health insurance accreditation in the three municipalities. The physicians’ exact locations are specified as part of the provided dataset (cf. Fig. 5) and the physicians opening hours were obtained from the Association of Statutory Health Insurance Physicians Nordrhein [37]. All considered physicians are closed on Saturdays and Sundays. Concerning the employed strategies, all physicians $\phi \in \mathcal {G}$ apply the individual-block/fixed-interval appointment scheduling strategy, priority first come, first served treatment strategy (PFCFS), and priority threshold admission strategy; cf. Appendix A.1.

#### Patients

The latest publicly available high resolution population data for the considered region is the German Census conducted in 2011 [33]. At a resolution of 2754 population cells measuring one hectare each, the 2011 Census reports a total population of 35542 for the three municipalities; compare Fig. 5. This population includes children under the age of 16 who the Census records on municipality level: Roetgen 1390, Simmerath 2383, and Monschau 1794. To exclude children under the age of 16 from our patient population, we proceed as follows: First, we fix one adult per population cell as we assume that children under the age of 16 do not live on their own. Then, we sample the number of under 16-year-olds from the remaining population of each municipality according to a uniform distribution.

Performing this procedure for each municipality individually, we obtain the final patient population $\mathcal {P}$ consisting of 29975 patient agents distributed over 2754 population cells.

The location $\ell \in {\mathscr{L}}$ for each patient is sampled from the associated population cell according to a uniform distribution. Patients’ health conditions *c* ∈ [0,1] are sampled from a beta distribution with shape parameters *p* = *q* = 25 such that all patients have an expected health condition of $\mathbb {E}(c)=0.5$.

#### Age classes

The baseline scenario differentiates three patient age classes: young (16-24), middle-aged (25-65), and elderly (> 65). The characteristics of the modeled age classes $\mathcal {A}$ are shown in Table 9.

**Table 9:** Age classes $\mathcal {A}$

	16-24	25-65	> 65
exp. illnesses	*I*_*a*_(*c*)= 6*c*	*I*_*a*_(*c*)= 7*c*+ 1	*I*_*a*_(*c*)= 9*c*+ 1
dev. duration	${\Delta }^{d}_{a}{=} 0.8$	${\Delta }^{d}_{a}{=}1.0$	${\Delta }^{d}_{a}{=}1.2$
dev. willingness	${\Delta }^{\omega }_{a} {=}1.2$	${\Delta }^{\omega }_{a} {=}1.0$	${\Delta }^{\omega }_{a} {=}0.8$
prob. cancel	*p*_*a*_= 0.95	*p*_*a*_= 0.8	*p*_*a*_= 0.7

Based on Census data [33], the age class $a\in \mathcal {A}$ of each patient depends on the discrete probability distribution shown in Table 10. The age-class-dependent attributes of each patient agent $\rho \in \mathcal {P}$ are subsequently determined as follows: Each patient’s session availabilities *α* are determined by performing a Bernoulli trial based on the age-class dependent success probabilities from Table 10. To decide whether a patient is chronically ill, we perform a Bernoulli trial using the success probabilities from Table 10 that were estimated based on [53].

**Table 10:** Age specific parameters for patient generation

	16-24	25-65	> 65
age class distribution	0.1196	0.6318	0.2486
availability probability	0.85	0.55	0.95
chronic illness probability	0.12	0.33	0.52

#### Families of illnesses

The most important classification system for illnesses world-wide is the International Classification of Diseases and Related health Problems (ICD) maintained by the World Health Organization. In its current revision, ICD-10 [3] distinguishes more than 14000 codes. For the purpose of SiM-Care, such a granular illness distinction is generally not necessary. Thus, we can aggregate ICD-10 codes, e.g., using the 22 chapters of ICD-10, or considering only a subset of all ICD-10 codes, e.g., the ones most frequently reported. In the baseline scenario, we consider a subset of the 100 ICD-10 codes most frequently reported to the Association of Statutory Health Insurance Physicians Nordrhein [38]. The attributes of families of illnesses can be estimated based on historical treatment data which is commonly available to health insurers. This data is protected by confidentiality and cannot be published. Thus, we only estimate all attributes which yields the families of illnesses $\mathcal {F}$ listed in Table 11.

**Table 11:** Characteristics of considered families of illnesses $f \in \mathcal {F}$

ICD	Name	Exp. willingness *W*_*f*_	Exp. duration *D*_*f*_	Treatment frequency *N*_*f*_	Is chronic
I10	high blood pressure	*W*_*f*_(*s*) = − 10*s* + 20	not applicable	*N*_*f*_(*s*) = − 20*s* + 100	true
E11	diabetes	*W*_*f*_(*s*) = − 4*s* + 14	not applicable	*N*_*f*_(*s*) = − 10*s* + 90	true
I25	ischemic heart disease	*W*_*f*_(*s*) = − 4*s* + 10	not applicable	*N*_*f*_(*s*) = − 30*s* + 100	true
E78	high cholesterol level	*W*_*f*_(*s*) = − 5*s* + 8	*D*_*f*_(*s*) = 4*s* + 8	*N*_*f*_(*s*) = − 2*s* + 11	false
M54	back pain	*W*_*f*_(*s*) = − 3*s* + 4	*D*_*f*_(*s*) = 9*s* + 5	*N*_*f*_(*s*) = − 4*s* + 11	false
Z25	vaccination	*W*_*f*_(*s*) = 40	not applicable	not applicable	false
J06	cold	*W*_*f*_(*s*) = − 2*s* + 2	*D*_*f*_(*s*) = 5*s* + 4	*N*_*f*_(*s*) = −*s* + 6	false

#### Age-class-illness distributions

The age-class-illness distribution $\pi ^{\text {act}}\colon \mathcal {A} \times \mathcal {F}^{\text {act}} \to [0,1]$ is estimated on the basis of the reported incidence rates of a large German health insurer published in [27]. We aggregate [27] by gender and age to obtain the age-class-illness distribution shown in Table 12. Analogously, we determine the expected distribution of chronic families of illnesses $\mathcal {F}^{\text {chro}}$ among the modeled age-classes $\mathcal {A}$ denoted by $\pi ^{\text {chro}}\colon \mathcal {A} \times \mathcal {F}^{\text {chro}} \to [0,1] $ shown in Table 12.

The distribution *π*^chro^ is not part of the baseline scenario, as it is only required to generate the unique chronic illness of chronic patients. This process is analogous to the process of generating acute illnesses described in Appendix A.3.

**Table 12:** Age-class-illness distributions *π*^act^ and *π*^chro^

	16-24	25-65	> 65
high cholesterol level	0.02	0.24	0.36
back pain	0.32	0.38	0.28
vaccination	0.14	0.14	0.27
cold	0.52	0.24	0.09
high blood pressure	0.17	0.65	0.61
diabetes	0.33	0.16	0.2
ischemic heart disease	0.5	0.19	0.19

#### Duration of warm-up

**Fig. 6:**
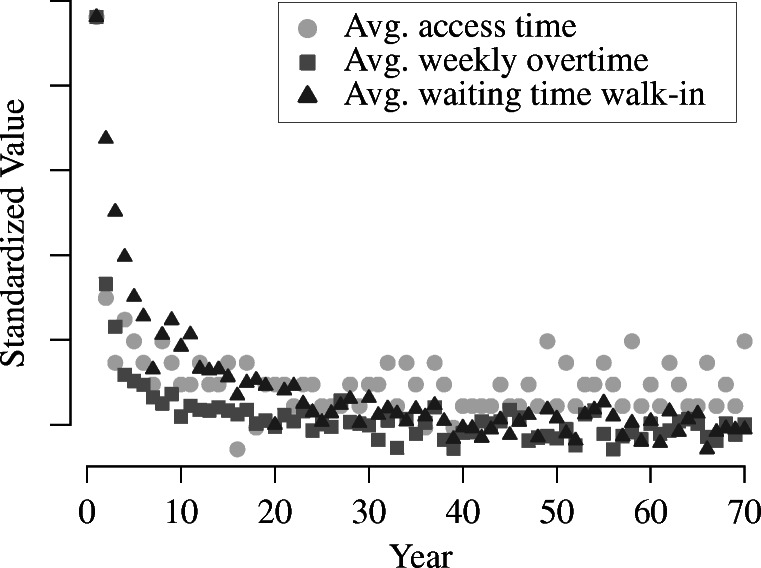
Data series of performance indicators in the baseline scenario for every year in a 70 year time period

To decide an appropriate length for the warm-up period, we simulate the baseline scenario for a time period of 70 years and track all performance indicators for each year individually. Figure 6 shows the resulting data series for the average access time, average weekly overtime, and average waiting time of walk-ins. The illustrated series suggest that the performance indicators stabilize after 20 to 40 years. To formalize this assessment, we employ the Schruben-Singh-Tierney test for initialization bias [60] to determine an appropriate truncation point for the warm-up period. Specifically, we use a two-sided test with a significance level of 5*%*. The scaling parameter is estimated using the batch means method with 5 batches based on the last half of the data series as suggested in [41]. Increasing the warm-up duration in steps of 5 years, the null-hypothesis of no initialization bias is first not rejected for all performance indicators for a warm-up duration of 30 years. Consequently, we choose a warm-up period of 30 years in the following.

### Baseline analysis

Table 13 reports the resulting expected key performance indicators as well as the associated exact 95 %-confidence intervals for each tracked performance indicator; compare Section 3.5. In the status quo, each physician performs, on average, 10137 treatments per year. This amounts to an average number of 6.76 physician contacts per patient, which is slightly above the 6.6 annual PCP contacts reported back in 2006 [1]. Roughly 47 % of patients visiting a physician in our baseline scenario are walk-in patients, which is consistent with the observed 48 % share of walk-ins in our empirical dataset of service times; compare Appendix A.3. We were unable to obtain empirical data on overtimes, as most primary care physicians are self-employed and even the definition of overtime is unclear. However, the estimated average overtime per physician (according to our definition) seems to be too low at just 4 minutes per week. This can be explained by the incorporated buffers and the exclusion of mandatory physician’s activities, such as reporting and accounting, from the simulation model.

**Table 13:** Mean performance indicators and 95 %-confidence intervals obtained by repeating each simulation experiment 20 times for each simulation scenario variant

	Baseline Scenario		Decline in PCPs Short-term Shift		Decline in PCPs Medium-term Shift		Aging Patients Short-term Shift	
	Mean	95 %-CI	Mean	95 %-CI	Mean	95 %-CI	Mean	95 %-CI
avg. # treatments	10137	[10128, 10146]	12444	[12431, 12457]	15094	[15085, 15104]	10237	[10229, 10245]
avg. # walk-ins	4747	[4738, 4756]	6967	[6954, 6980]	9449	[9440, 9459]	4846	[4838, 4854]
avg. # acute appts.	3214	[3212, 3216]	2764	[2761, 2768]	2331	[2324, 2339]	3192	[3191, 3193]
avg. # regular appts.	2176	[2175, 2178]	2713	[2709, 2716]	3313	[3306, 3321]	2199	[2198, 2200]
avg. utilization [%]	72	[72, 72]	81	[81, 81]	89	[89, 89]	73	[72, 73]
avg. weekly overtime [min]	4	[4, 4]	15	[15, 16]	58	[57, 60]	4	[4, 4]
avg. # rejected walk-ins	17	[16, 18]	91	[88, 93]	473	[463, 482]	17	[16, 18]
avg. access time [d]	2.5	[2.5, 2.5]	3.2	[3.2, 3.2]	4.1	[4.1, 4.2]	2.5	[2.5, 2.5]
avg. access time regular [d]	1.5	[1.5, 1.5]	1.6	[1.6, 1.6]	1.9	[1.8, 1.9]	1.5	[1.5, 1.5]
avg. access distance [km]	4.8	[4.8, 4.8]	6	[6.0, 6.0]	7.2	[7.2, 7.3]	4.8	[4.8, 4.8]
avg. waiting time appt. [min]	2	[2, 2]	2	[2, 2]	2	[2, 2]	2	[2, 2]
avg. waiting time walk-in [min]	40	[40, 40]	52	[52, 52]	67	[67, 67]	40	[40, 41]
on-time appts. [%]	61	[61, 61]	59	[59, 59]	59	[58, 59]	61	[61, 61]
# acute illnesses	136405	[136234, 136575]	136553	[136386, 136720]	136344	[136227, 136460]	137731	[137554, 137908]
# chronic patients	10662	–	10662	–	10662	–	10776	–
total PCP capacity [h]	32617	–	26455	–	22139	–	32617	–
	Aging Patients Medium-term Shift		Combined Effects Short-term Shift		Combined Effects Medium-term Shift			
	Mean	95 %-CI	Mean	95 %-CI	Mean	95 %-CI		
avg. # treatments	10325	[10313, 10337]	12579	[12567, 12591]	15368	[15356, 15380]		
avg. # walk-ins	4934	[4921, 4946]	7102	[7090, 7114]	9723	[9711, 9734]		
avg. # acute appts.	3160	[3158, 3162]	2741	[2736, 2745]	2258	[2251, 2264]		
avg. # regular appts.	2231	[2229, 2233]	2736	[2732, 2741]	3388	[3381, 3395]		
avg. utilization [%]	73	[73, 73]	81	[81, 81]	90	[90, 90]		
avg. weekly overtime [min]	4	[4, 5]	17	[16, 18]	67	[66, 68]		
avg. # rejected walk-ins	20	[19, 21]	100	[97, 103]	560	[548, 572]		
avg. access time [d]	2.6	[2.6, 2.6]	3.3	[3.3, 3.3]	4.4	[4.4, 4.4]		
avg. access time regular [d]	1.5	[1.5, 1.5]	1.7	[1.6, 1.7]	2	[1.9, 2.0]		
avg. access distance [km]	4.9	[4.9, 4.9]	6.1	[6.1, 6.1]	7.3	[7.3, 7.3]		
avg. waiting time appt. [min]	2	[2, 2]	2	[2, 2]	2	[2, 2]		
avg. waiting time walk-in [min]	41	[40, 41]	53	[53, 53]	68	[68, 68]		
on-time appts. [%]	61	[61, 61]	59	[59, 59]	59	[58, 59]		
# acute illnesses	138696	[138478, 138913]	137886	[137686, 138086]	138595	[138468, 138722]		
# chronic patients	10931	–	10776	–	10931	–		
total PCP capacity [h]	32617	–	26455	–	22139	–		

Patients in the baseline scenario are expected to travel almost 5 km to visit a physician and have to wait an average 2.5 days for their appointments. With regard to waiting times, we obtain an average expected waiting time of 2 minutes for patients with appointment and 40 minutes for walk-in patients. In comparison to the average waiting times observed when recording our service time dataset (4 minutes with appointment, 15 minutes without appointment), simulated waiting times are strikingly unfavorable for walk-in patients. We take this to suggest that physicians in the real world avoid excessive waiting times for walk-in patients through more sophisticated treatment strategies like [62].

### Scenario 1: Decline in PCPs

Scenario 1 models a decline in the number of PCPs for a short- and a medium-term shift in time. To that end, we exclude those PCPs from the baseline PCP population $\mathcal {G}$ that reached the statutory retirement age of 65 by this point. Specifically, we consider the year 2023, by which 4 out of 20 PCPs will reach the statutory retirement age, and the year 2027, by which 7 out of 20 PCPs will reach the statutory retirement age. Assuming that none of the excluded physicians are replaced by a successor, we obtain a decimated population of primary care physicians $\mathcal {G}^{\text {s}}$ for the short-term and $\mathcal {G}^{\text {m}}$ for the medium-term shift. By replacing the physician population $\mathcal {G}$ in our baseline scenario by $\mathcal {G}^{\text {s}}$ and $\mathcal {G}^{\text {m}}$, respectively, we obtain two variants for Scenario 1; see Table 14.

**Table 14:** Populations in each simulation scenario variant

	Scenario 1		Scenario 2		Scenario 3	
	s	m	s	m	s	m
patients	$\mathcal {P}$	$\mathcal {P}$	$\mathcal {P}^{\text {s}}$	$\mathcal {P}^{\text {m}}$	$\mathcal {P}^{\text {s}}$	$\mathcal {P}^{\text {m}}$
physicians	$\mathcal {G}^{\text {s}}$	$\mathcal {G}^{\text {m}}$	$\mathcal {G}$	$\mathcal {G}$	$\mathcal {G}^{\text {s}}$	$\mathcal {G}^{\text {m}}$

s = short-term shift, m = medium-term shift

The simulation results for Scenario 1 in Table 13 show a severe deterioration of all patient and physician indicators compared to the baseline scenario. The physicians’ expected workload measured through the average number of treatments increases by 23 % for the short-term and 49 % for the medium-term shift. Due to the increased scarcity of appointments, more and more patients are forced to visit physicians as walk-in patients (56 % for short-term and 63 % for medium-term shift). The average weekly overtime for physicians increases by 11 minutes for the short-term and 54 minutes for the medium-term shift. On average, patients wait 30 % longer for their appointments in the short-term and even 67 % longer in the medium-term shift scenario variant. Similar increases can be observed for the patients’ average access distance, which increases by 26 % for the short-term and by 51 % for the medium-term shift. The average waiting time for patients with appointment is almost unaffected by the decline in the number of physicians, which can be explained by the strict prioritization in PFCFS. The average waiting time for walk-in patients increases by 30 % for the short-term and 66 % for the medium-term shift.

### Scenario 2: Aging patients

Scenario 2 models the ongoing aging of the patient population for a short- and medium-term shift in time. For this purpose, we adjust the discrete probability distribution determining the patients’ age classes to generate two new patient populations. More precisely, we use current population projections [34] for the years 2025 and 2030 to obtain the two adjusted discrete probability distributions in Table 15. Using these distributions, we generate the aged patient population $\mathcal {P}^{\text {s}}$ for the short-term and $\mathcal {P}^{\text {m}}$ for the medium-term shift. By replacing the patient population $\mathcal {P}$ in our baseline scenario by $\mathcal {P}^{\text {s}}$ and $\mathcal {P}^{\text {m}}$, respectively, we obtain two scenario variants for Scenario 2; compare Table 14.

**Table 15:** Age class distributions for aged patient population

	16-24	25-65	> 65
short-term shift	0.1051	0.6283	0.2666
medium-term shift	0.1025	0.6033	0.2942

The simulation results for Scenario 2 (Table 13) paint a similar picture as in Scenario 1, i.e., the majority of patient and physician indicators deteriorate, albeit far less severely. The average number of treatments per physician increases by 1 % for the short-term and by 2 % medium-term shift. In contrast to Scenario 1, additional treatments distribute more evenly across appointment and walk-in patients and thus the expected ratio of walk-in patients remains almost unchanged. Physicians seem to accommodate the additional treatments within their regular opening hours, as their average overtime is almost unaffected. Due to the increased demand, patients wait on average 2 % longer for their appointments in the short-term and 5 % longer in the medium-term shift variant. Moreover, they are willing to accept 1 % longer average access distances in both scenario variants. Patient waiting times with appointment are unaffected by the increased demand. The average waiting times of walk-in patients in the short-term shift remain almost unchanged, while they increase by 1 % for the medium-term shift.

### Scenario 3: Combined effects

Scenario 3 models a combined decline in the number of PCPs and aging of the patient population for a short- and medium-term shift. By replacing both populations from the baseline scenario with the adjusted patient and physician populations from Scenarios 1 and 2, we obtain two variants for Scenario 3; compare Table 14.

Analyzing the simulation results in Table 13 for Scenario 3 confirms that the combined effects lead to the greatest deterioration of indicators. The effect of the combined changes compared to the combination of effects from Scenarios 1 and 2 varies between indicators: For the average number of treatments and the ratio of walk-in patients, the effects of the combined changes correspond to the sum of the effects for the individual changes, e.g., a 24 % increase in the average number of treatments in short-term shift variant of Scenario 3 versus a 23 % and 1 % increase in the respective variants of Scenarios 1 and 2. For the physicians’ average overtime, a combined consideration of both changes has an amplifying effect. For example, in the medium-term shift variants of Scenarios 1 and 2 the average overtime increases by 54 and 0 minutes, respectively, while the combined changes in Scenario 3 lead to an increase of 63 minutes. Similar amplifying effects can be observed for the patients’ average access time, access distance, and walk-in waiting time.

### Sensitivity analysis

SiM-Care’s complexity and large number of input parameters make the model versatile, but also create the risks of instability and high sensitivity towards small changes in the input values. To ensure that such undesired behaviors do not invalidate the outputs of simulation experiments, we perform a sensitivity analysis.

The sensitivity analysis considers the baseline scenario in the setup of the case study, i.e., 20 independent runs modeling one year preceded by a warm-up of 30 years. As the complexity of the model prohibits a full sensitivity analysis within the scope of this paper, we demonstrate the process for those input parameters from Table 8 that are least anchored in empirical data. Specifically, we study the model’s sensitivity towards the patients’ health condition *c* ∈ [0,1] and the age classes’ deviation from the illness duration ${\Delta }^{d}_{a} \geq 0$, deviation from the willingness to wait ${\Delta }^{\omega }_{a} \geq 0$, and probability to cancel an appointment after recovery *p*_*a*_ ∈ [0,1].

**Fig. 7:**
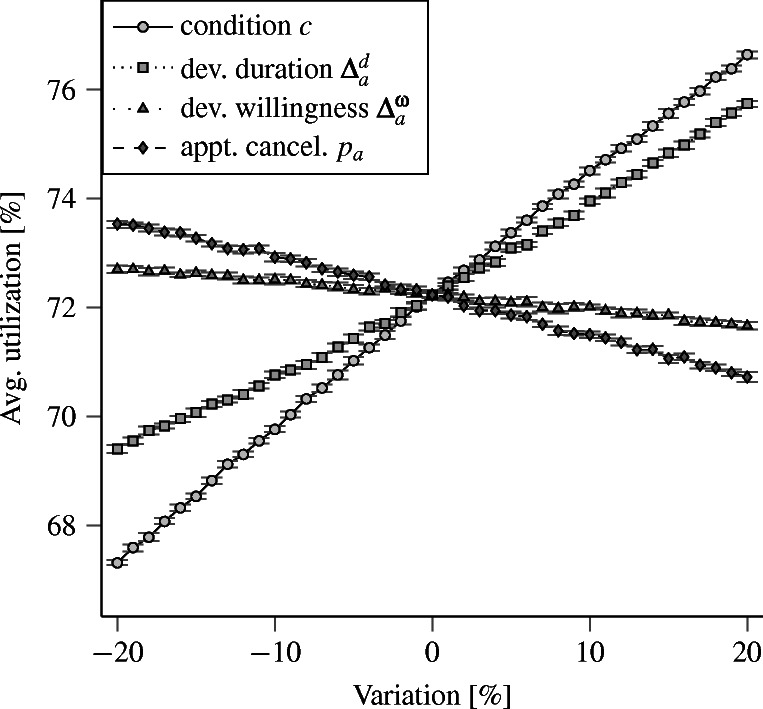
Mean average utilization and corresponding 95 % exact confidence intervals

**Fig. 8:**
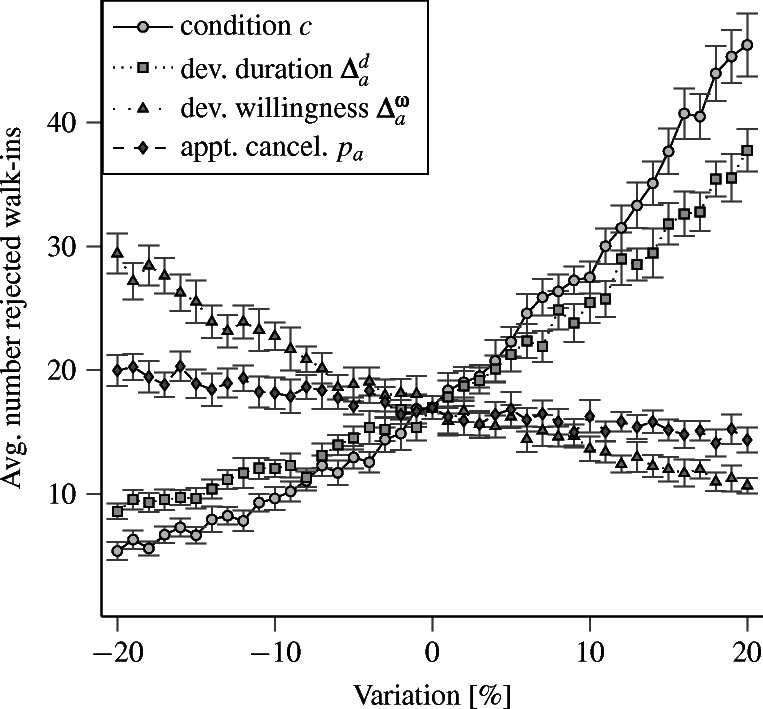
Mean average number of rejected walk-in patients and corresponding 95 % exact confidence intervals

We vary each input parameter relative to its original value between ± 20 % in increments of 1 %. We analyze the resulting impact on the PCPs’ average utilization and number of rejected walk-in patients. Figures 7 and 8 show the resulting average values and 95 % confidence intervals for both performance indicators.

These results do not indicate that the complexity of SiM-Care causes instabilities. Instead, both performance indicators behave as expected towards variations of the input parameters, e.g., increasing the patients’ deviation from the willingness to wait ${\Delta }^{\omega }_{a} \geq 0$ causes patients to wait longer for appointments, which in turn reduces treatments and thus decreases utilization and rejected walk-ins.

The shape of the relationship between changes in input and output reveals two phenomena. For the average utilization shown in Fig. 7, the relationships is almost linear with small confidence intervals. Moreover, the slopes within these relationships are relatively flat, which indicates a low sensitivity towards small changes in the input values. For the average number of rejected walk-in patients (Fig. 8), the relationship is non-linear with large confidence intervals. The sensitivity and the variation in the number of rejected walk-in patients furthermore increase with the system’s utilization, which seems intuitive. Still, for small changes (± 5 %) of the considered input values, the sensitivities are not extreme given the width of the confidence intervals.

## Conclusion and future work

SiM-Care aims to support decision makers in planning, analyzing, and adapting primary care systems. It produces meaningful performance indicators that enable a far more detailed assessment of primary care systems compared to the current approaches based on patient-physician ratios. SiM-Care can predict and quantify the influence of policy decisions and changes in the systems population, e.g., an aging of the population or a decline in the number of PCPs as illustrated in Section 4. Because SiM-Care can particularly model multiple simultaneous system changes, it enables the analysis of combined effects. As all components of a simulation scenario can be easily adjusted, this opens up a broad field of potential applications ranging from physicians’ location planning to the evaluation of specific PCP strategies, e.g., in the field of appointment scheduling. However, as pointed out in [68], we must note that the resulting predictions can never be completely accurate and are therefore intended to scan the horizon and “inform policy makers about future problems and development”. Finally, the modular design of SiM-Care and its planned availability as open source code allows for easy model extensions, e.g., to model prospective new supply concepts such as mobile medical units or telemedicine. Moreover, one could also change the setup of simulation experiments, e.g., by using the batch means method instead of independent runs to trade robustness for computational efficiency; compare [42].

The greatest challenge to using SiM-Care in practice is the complex and time consuming task of generating and validating the input scenarios. As SiM-Care models each agent individually, it requires detailed empirical data, which has to be obtained from various parties or may be unavailable. Additionally, even the estimation of parameters from empirical data is generally a challenge on its own that we neglected in this work. The current version of the system tailors some model components such the service time distributions to the German system. These components might have to be adjusted when using SSiM-Care to analyze, e.g., a primary care system in the United States. Such model changes potentially change the model’s behavior and thus require a new validation process to ensure that insights derived from SiM-Care are viable.

To help overcoming this challenge, we exemplified the scenario generation and validation process for a real-world primary care system. Particularly, we detailed the generation process of all simulation entities and provided available empirical data sources. Although data availability may vary for other primary care systems, this may hint at where the required empirical data can be obtained. We validated our simulation scenario by comparing its output to available empirical data. To show internal validity, we captured the model variability through confidence intervals. However, we need to stress that additional validation should be performed before actual policy decisions are derived from the presented case study. Such validation measures should particularly include an expert validation which was out of scope for the purpose of this study.

Conceptual modeling needs to be judged by validity, feasibility, utility, and credibility [54, 55]. So far, we discussed validity at length and demonstrated a degree of feasibility and utility in our case study. However, trust can not come from validation alone, but must also rely on the credibility of modelers and stakeholders [30]. Thus, we conclude by turning to the subject of credibility.

Credibility can only be given from the client’s perspective and requires stakeholder involvement and case studies, as emphasized in [63]. For a stakeholder definition and consideration of diverse stakeholder groups, we rely on [12]. Based on flow charts and early prototypes of SiM-Care, we discussed the conceptual model and experimental designs with professionals (PCPs), health care providers, and a municipal government agency. However, up to the time of this writing, the project neglected, i.a., patient interest groups. Furthermore, we did not implement a systematic framework for stakeholder involvement as proposed, e.g., in [44, 63]. One reason for this was that SiM-Care resulted from a project aiming to quantify the effects of new mathematical planning approaches, e.g., to schedule patient appointments. Therefore, the related project constituted what [12] term “curiosity-driven academic research” rather than practice-based research.

Some arguments on generality and agility speak against a strictly sequential development involving stakeholders such as the PartiSim framework introduced in [63]: As [63] point out, the health care domain is driven by “many decision-makers” and “busy stakeholders”. At the same time, SiM-Care aims to support applications from “horizon scanning” (cf. [68]) to evaluate the impact of changing specific submodels, e.g., a PCP’s approach to scheduling patients. We consider it as highly challenging to elicit views and involvement from a sufficiently broad selection of decision-makers and stakeholders to create such a generally applicable simulation model. Even more importantly, an agile rather than sequential view of developing simulation systems may reduce the risk of project failure; compare [58].

Agile development does not strictly adhere to a process that lays down the conceptual model before the coding phase. Instead, it does allow for adjusting the model even in the experimentation phase. To use an agile approach to development, we suggest a systematic stakeholder involvement in the post-model-coding-stages as exemplified in [43]. In other words, we recommend emphasizing stakeholder involvement more strongly when designing case studies that rely on SiM-Care.

Finally, to implement more stakeholder-driven research means working to overcome the challenges of stakeholder “identification, contact, and elicitation” [12]. Somewhat paradoxically, in doing that, having implemented and published research with the current version of SiM-Care might help engagement. This did appear to be the case when presenting SiM-Care to the general public at a university outreach event in 2019. During this event, participants could generate appointment scheduling rules and discuss the effects of their ideas as computed by the simulation. As suggested in [43], a pick-and-mix approach might support such a view and enable facilitated simulation modeling, coding, and experimentation.

Future work on SiM-Care will include further effort towards model validation and calibration as well as the implementation model extensions. Currently, illness distributions are considered as being static by SiM-Care. By modeling dynamic illness distributions, we can incorporate seasonality or the patients’ previous history of illnesses. In the current model, the duration of an illnesses is independent of the actual treatment. Interestingly, the results are convincing even without this causal link. In the future, we want to compare whether implementing this link in the conceptual model significantly affects our findings. A similar comparison shall investigate the influence of no-show patients, who introduce unexpected idle time into the physicians’ schedules. While the sole integration of patient non-attendance into SiM-Care is straightforward, the actual difficulty lies in the need for empirical no-show probabilities as well as the necessity to decide how no-show patients continue their course of treatment. Yet other possible model extensions include: Illness specific appointments, intentional physicians’ breaks, implementation of additional patient attributes such as gender, and mobile patient agents that move between different locations, e.g., their home and work. Finally, we are currently preparing the open source release of our model implementation that comes with a graphical user interface such that SiM-Care can be easily accessed, studied, and adapted to the individual requirements of all modelers.

## Figures and Tables

**Fig. 9:**
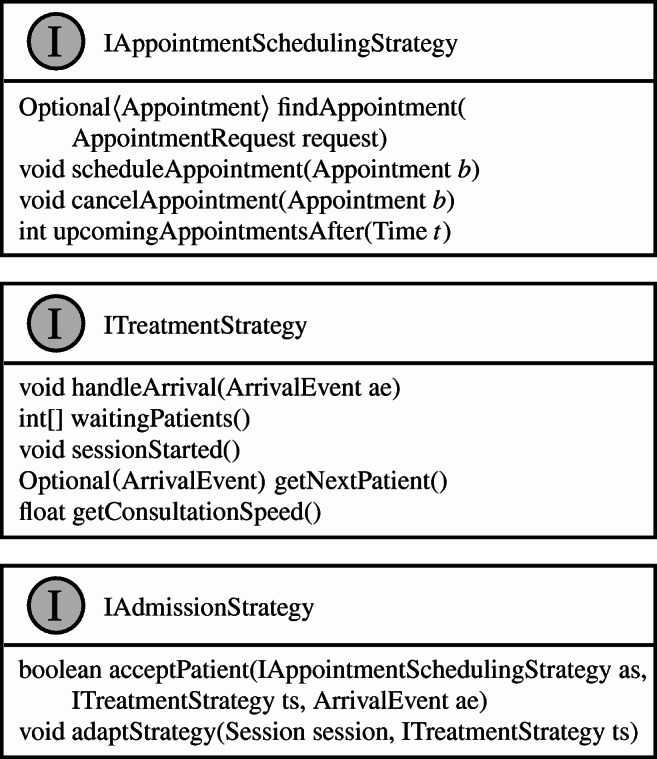
Interfaces implemented by strategies

**Fig. 10:**
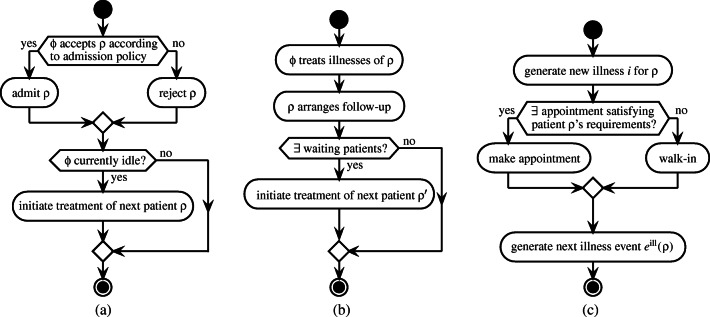
Processing of **a** arrival events *e*^arv^(*ϕ*, *ρ*), **b** release events *e*^rel^(*ϕ*, *ρ*), and **c** illness events *e*^ill^(*ρ*); $\rho \in \mathcal {P}$ and $\phi \in \mathcal {G}$

**Fig. 11:**
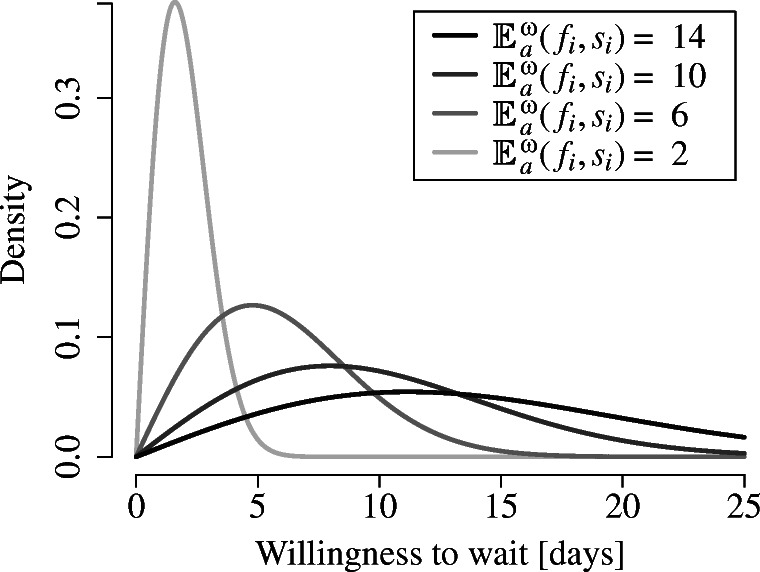
Weibull distributions of $\omega _{i}\in \mathcal {T}$ for different values of patient’s age adjusted expected willingness to wait $\mathbb {E}^{\omega }_{a}(f_{i},s_{i})$

**Fig. 12:**
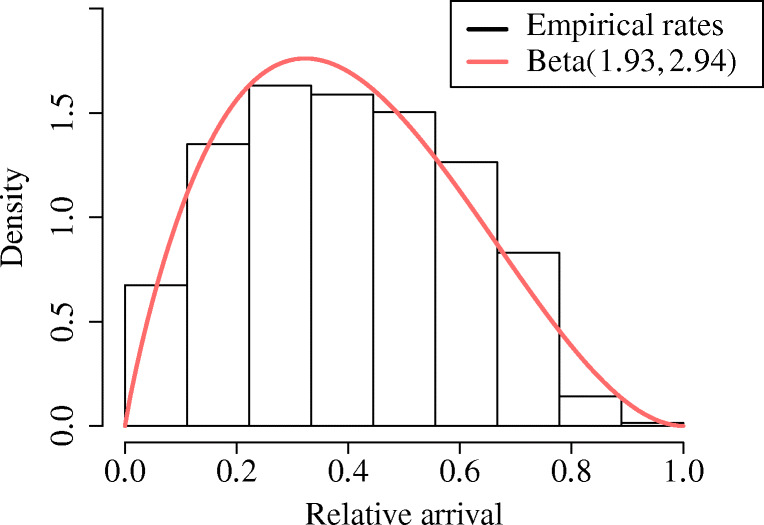
Histogram and beta distributed maximum-likelihood fit for empirical walk-in arrival rates from [66]

**Fig. 13:**
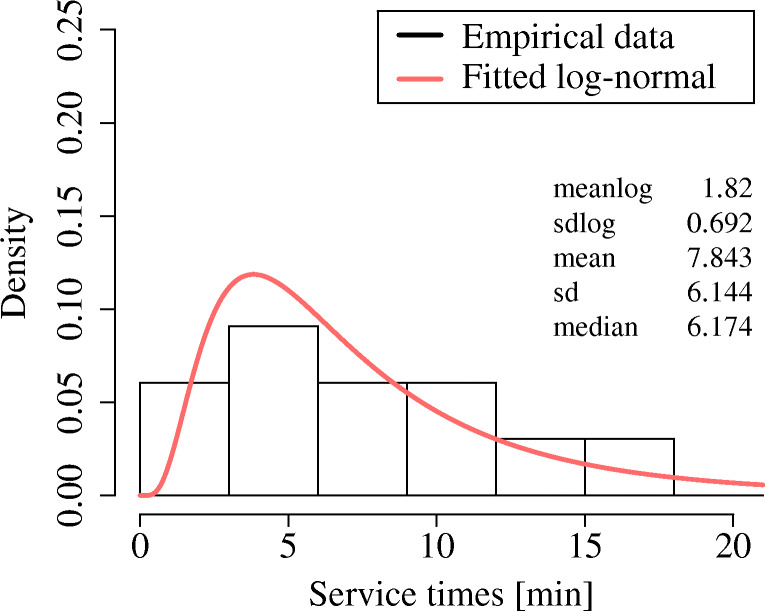
Histogram and log-normal maximum-likelihood fit for empirical service times of patients with appointment

**Fig. 14:**
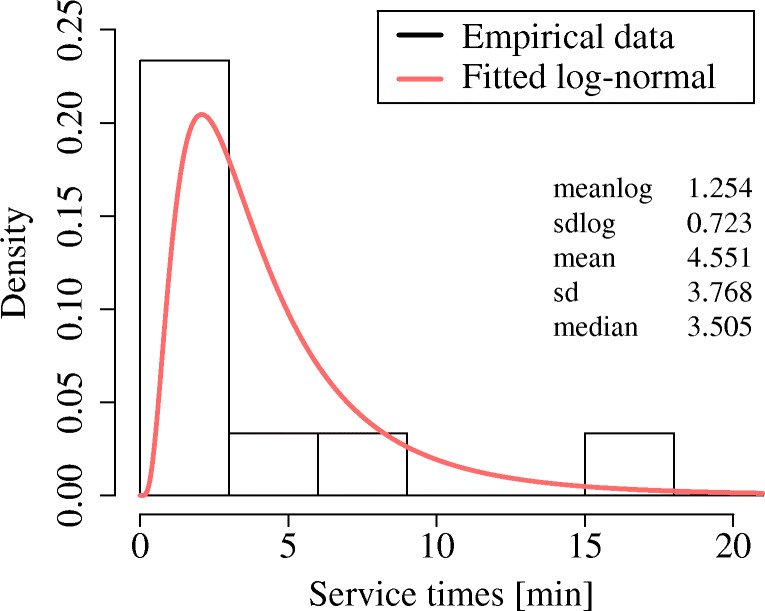
Histogram and log-normal maximum-likelihood fit for empirical service times of walk-in patients

## Appendix A

### A.1 PCP strategies

The feasible set of appointment scheduling strategies $\mathcal {S}^{\text {app}}$ is defined via the interface shown in Fig. 9. That is, every appointment scheduling strategy $S\in \mathcal {S}^{\text {app}}$ has to provide the functionality to answer appointment requests with an appointment suggestion (that can be empty). Thereby, every appointment request specifies the requesting patient, earliest possible appointment time, willingness to wait, whether the request is for a regular appointment, and whether patient’s availabilities have to be respected. Furthermore, every appointment scheduling strategy $S\in \mathcal {S}^{\text {app}}$ has to provide the functionality to schedule previously offered appointments as well as the functionality to cancel previously scheduled appointments. Finally, every appointment scheduling strategy $S\in \mathcal {S}^{\text {app}}$ has to be able to compute the number of upcoming appointments within a session that are scheduled to take place after a specified point in time.

The *Individual-block/ Fixed-interval* (IBFI) appointment scheduling strategy evenly spaces out appointments throughout each session; see [17, 40]. To that end, it divides the opening hours of each session in a 140 day rolling horizon into slots of 15 minutes length. Each slot can accommodate one appointment and slots are offered to patients on a first-come-first-served (FCFS) basis. Thus, no appointments are withheld and every patient is offered the earliest feasible appointment at the time of inquiry.

The feasible set of treatment strategies $\mathcal {S}^{\text {tmt}}$ is defined via the interface shown in Fig. 9. That is, every treatment strategy $S\in \mathcal {S}^{\text {tmt}}$ has to keep track of admitted patients, count the number of waiting patients with and without appointment, and define how the treatment strategy is affected by the beginning of a session. Moreover, every treatment strategy $S\in \mathcal {S}^{\text {tmt}}$ has to determine the next patient to be treated (that might not exist) as well as the PCP’s current consultation speed which is thoroughly discussed in Appendix A.6.

The *priority first come, first served* (PFCFS) treatment strategy is popularly used in studies of health systems [17]. In PFCFS, patients with appointment are prioritized over walk-ins and within their respective groups, patients are served in order of their arrival, i.e., FCFS; compare [19, 52]. Patients that arrive before the beginning $\underline {o}(\lambda ) \in \mathcal {T}$ of session *λ* ∈Λ have to wait and the physician does not start treatments until the session has officially begun. The PCP’s standard consultation speed in PFCFS is *ζ* = 1.0, which is adjusted to *ζ* = 0.8 whenever more than 3 patients await treatment; compare Appendix A.6.

The feasible set of admission strategies $\mathcal {S}^{\text {adm}}$ is defined via the interface shown in Fig. 9. That is, every admission strategy $S\in \mathcal {S}^{\text {adm}}$ has to be able to decide whether an arriving patient is admitted or not given the PCP’s treatment and appointment scheduling strategy. Moreover, every admission strategy $S\in \mathcal {S}^{\text {adm}}$ may define adaptive traits that allow PCPs to learn and adapt their treatment strategy.

The *priority threshold* (PT) admission strategy admits patients up to a certain utilization threshold; compare [39, 51]. PT differentiates between appointment, walk-in, and emergency patients: Emergency patients are always admitted. Patients with an appointment in session *λ* ∈Λ are admitted as long as their time of arrival $t^{\text {arr}}\in \mathcal {T}$ is before the end of the session’s buffer, i.e., $t^{\text {arr}} \leq \overline {o}(\lambda ) + \frac {1}{24}$. For the admittance of walk-in patients, physicians predict their remaining workload by multiplying an expected service time with the number of currently waiting patients and upcoming scheduled appointments. If this estimated workload is lower than the remaining duration of the current session including buffer, walk-in patients are admitted, otherwise rejected. The expected service time is initialized to 7 min and adjusted at the end of each session as follows. If three or more patients are awaiting treatment at the end of the anticipated buffer, the expected service time is increased by 1 min. If the physician is idle at the end of the buffer although walk-ins were rejected, the expected service time is reduced by 20 sec.

### A.2 Events

*Arrival events* are indicated by *e*^arv^(*ϕ*, *ρ*). As illustrated in Fig. 10a, they mark the event of patient *ρ* arriving at physician *ϕ*’s practice for an appointment or as a walk-in. The physician’s decision to admit or reject arriving patients depends on *ϕ*’s admission strategy. Every admitted patient is guaranteed to receive treatment and enters the physician’s waiting room. If the physician is currently idle, this triggers the physician’s treatment strategy and treatment commences.

*Follow-up events* are indicated by *e*^fol^(*ϕ*, *ρ*, *i*). Some families of illnesses $f_{i} \in \mathcal {F}$ cannot be treated via a single visit. Instead, the related illnesses $i\in \mathcal {I}$ require follow-up treatments in intervals defined by the parameter *ν*_*i*_≠*∅*. Ensuring continuous follow-up treatments, patients always try to arrange a follow-up appointment immediately after the treatment of illnesses requiring follow-up consultation. To account for the fact that no feasible follow-up appointment might be available, SiM-Care generates a follow-up event *e*^fol^(*ϕ*, *ρ*, *i*) at time *t*^treat^ + *ν*_*i*_ every time illness $i\in \mathcal {I}$ with *ν*_*i*_≠*∅* suffered by patient $\rho \in \mathcal {P}$ is treated by physician $\phi \in \mathcal {G}$ at time $t^{\text {treat}}\in \mathcal {T}$. Follow-up events serve as the patient’s reminder to actively re-pursue follow-up consultation for illness *i* after the duration of the follow-up interval. Triggered by a follow-up event *e*^fol^(*ϕ*, *ρ*, *i*), patient *ρ* reattempts to arrange a follow-up appointment with physician *ϕ*. Should *ϕ* once again be unable to provide a suitable appointment, *ρ* seeks follow-up consultation as a walk-in patient. Every follow-up treatment of an illness $i\in \mathcal {I}$ invalidates all associated existing follow-up events, as the follow-up interval is reset.

As a result, follow-up events only trigger if an illness has not been treated for the duration of its follow-up interval $\nu _{i}\in \mathcal {T}$.

*Release events* are indicated by *e*^rel^(*ϕ*, *ρ*). As illustrated in Fig. 10b, release events mark the event of physician *ϕ* releasing patient *ρ* after a treatment is performed. Whenever a new treatment begins, the sampled service time determines the time of the subsequent release event *e*^rel^(*ϕ*, *ρ*). All treated illnesses $i\in \mathcal {I}^{\text {act}}$ without duration (*d*_*i*_ = *∅*) are cured through a one-time treatment and thus removed from $\mathcal {I}^{\text {act}}$. Subsequently, all existing follow-up events corresponding to treated illnesses are deleted and new follow-up events are generated in the previously described manner. The successful treatment revokes emergency flags. If the patient’s chronic illness ${\varsigma } \in \mathcal {I}$ was treated, the next recurrent regular appointment $b^{\text {reg}}\in {\mathscr{B}}$ with physician *ϕ*^fam^ is requested at time *t*^treat^ + *ν*_*ς*_. Then, patients request an acute appointment $b^{\text {act}}\in {\mathscr{B}}$ with physician *ϕ* for the follow-up treatment of the persisting acute illness $i^{*} = \text {argmin}_{i \in \mathcal {I}^{\text {act}} : \nu _{i}\neq \emptyset } \nu _{i}$ with smallest follow-up interval. The requested appointments ensure the follow-up treatment of all illnesses suffered by patient *ρ* and will preempt the previously generated follow-up events. Finally, physicians implement their treatment strategy to select the next patient from the waiting room if the latter is non-empty. Otherwise, physicians remains idle until the next arrival event triggers the treatment strategy. As a result of this behavior, physicians are never intentionally idle.

*Illness events* are indicated by *e*^ill^(*ρ*). As illustrated in Fig. 10c, they describe that patient *ρ* starts to suffer from a new acute illness. This means that the model generates a new acute illness $i\in {\mathscr{I}}^{\text {act}}$ with stochastic qualities that depend on the patient’s age and health condition and adds it to their set of illnesses $\mathcal {I}$. To treat the emerged illness, patients request an appointment from their preferred physicians or, in case this does not succeed, directly visit the preferred physician as a walk-in. As a result, each illness event generates a corresponding arrival event *e*^arv^(*ϕ*, *ρ*) and adds it to the queue $\mathcal {Q}$. Finally, each illness event generates a future illness event *e*^ill^(*ρ*) for patient *ρ* and adds it to the queue $\mathcal {Q}$ to mark the next point in time patient *ρ* develops an acute illness.

*Recovery events* are indicated by *e*^rec^(*ρ*, *i*). They mark the event of patient *ρ* recovering from acute illness $i\in \mathcal {I}^{\text {act}}$. Whenever the model generates a new acute illness $i\in {\mathscr{I}}^{\text {act}}$ with *d*_*i*_≠*∅*, it also generates a corresponding recovery event *e*^rec^(*ρ*, *i*) at time *t*^ill^ + *d*_*i*_, where $t^{\text {ill}}\in \mathcal {T}$ is the point in time illness *i* is developed. Illnesses without duration (*d*_*i*_ = *∅*) do not require a recovery event as they are immediately cured through their initial treatment. A recovery event removes illness *i* from $\mathcal {I}$ and deletes any associated follow-up event $e^{\text {fol}}(\phi , \rho , i)\in \mathcal {Q}$. If patient *ρ* does not suffer from acute illnesses following the removal of illness *i*, i.e., $\mathcal {I}^{\text {act}} = \emptyset $, the model revokes existing emergency flags and assumes that *ρ* may cancel scheduled acute appointments. Such cancellations occur with the patient’s age-class-specific probability *p*_*a*_ ∈ [0, 1] and consequently delete the associated arrival event *e*^arv^(*ϕ*, *ρ*). As a result, some patients keep their existing acute appointment for a final debriefing. Should patient *ρ* be currently seeking walk-in treatment due to persisting chronic illness ${\varsigma }\in \mathcal {I}$, this effort is continued. Otherwise, current walk-in attempts are canceled and the associated arrival event *e*^arv^(*ϕ*, *ρ*) is deleted.

*Open- and close events* are indicated by *e*^opn^(*ϕ*) and *e*^clo^(*ϕ*), respectively. They mark the beginning and ending (including buffer) of a session *λ* ∈Λ operated by physician *ϕ*. They ensure that treatment strategies become aware of a session’s beginning and that overtime is incurred for all treatments performed beyond the anticipated buffer time.

### A.3.1 Frequency of acute illnesses

The occurrence of acute illnesses in SiM-Care is modeled via a Poission process. Patients develop acute illnesses at a frequency that depends on their age and health condition. For patients $\rho \in \mathcal {P}$ of age class $a \in \mathcal {A}$ with health condition *c* ∈ [0, 1], the expected number of acute illnesses per year is given by the parameter *I*_*a*_(*c*). The intensity (or rate) of the Poission proccess is thus *I*_*a*_(*c*)/364 per day. Moreover, the duration between two consecutive illness events *e*^ill^(*ρ*) for patient *ρ* can be sampled from an exponential distribution with rate *I*_*a*_(*c*)/364 [21, chapter 2].

### A.3.2 Type of acute illnesses

Whenever an illness event *e*^ill^(*ρ*) occurs and patient $\rho \in \mathcal {P}$ falls ill, the model generates an acute illness $i\in {\mathscr{I}}^{\text {act}}$ according to the patients’ age class $a \in \mathcal {A}$ and health condition *c* ∈ [0, 1]. The model assumes a probabilistic link between illness family $f_{i}\in \mathcal {F}^{\text {act}}$ and the patient’s age class *a* that is expressed via the age-class-illness distribution *π*^act^; see Section 3.2.5. To that end, any emerging acute illness of patient *ρ* is randomly assigned to an illness family according to the discrete probability distribution *f*↦*π*^act^(*a*, *f*) for $f\in \mathcal {F}^{\text {act}}$.

### A.3.3 Qualities of acute illnesses

For any new illness $i\in {\mathscr{I}}^{\text {act}}$ of family $f_{i}\in \mathcal {F}$ generated through SiM-Care, its seriousness *s*_*i*_ ∈ [0, 1] depends on a triangular distribution defined on the closed interval [0, 1]. The distribution’s mode is the health condition *c* ∈ [0, 1] of patient $\rho \in \mathcal {P}$ developing illness *i*. Thus, patients with a bad health condition tend to develop more serious illnesses.

The duration $d_{i}\in \mathcal {T}$ of illness *i* depends on a log-normal distribution. Given *i*’s family of illnesses $f_{i}\in \mathcal {F}$, seriousness *s*_*i*_ ∈ [0, 1], and the patient’s age class $a\in \mathcal {A}$, we define the age-adjusted expected duration of illness *i* as $\mathbb {E}^{d}_{a}(f_{i},s_{i}):= {\Delta }^{d}_{a} \cdot D_{f_{i}}(s_{i})$. Therefore, SiM-Care samples the illness’ duration *d*_*i*_ from a log-normal distribution with sdlog *σ* = 0.3 and meanlog $\mu = \log (\mathbb {E}^{d}_{a}(f_{i},s_{i}))- \sigma ^{2}/2$.

The willingness to wait specified by $\omega _{i} \in \mathcal {T}$ depends on a Weibull distribution. Given *i*’s illness family $f_{i}\in \mathcal {F}$, seriousness *s*_*i*_ ∈ [0, 1], and the patient’s age class $a\in \mathcal {A}$, the age-adjusted expected willingness to wait of illness *i* is defined as $\mathbb {E}^{\omega }_{a}(f_{i},s_{i}):= {\Delta }^{\omega }_{a} \cdot W_{f_{i}}(s_{i})$. Analogously to Wiesche et. al [67], we sample *ω*_*i*_ from a Weibull distribution with shape parameter *p* = 2 and derive the scale parameter from the age adjusted expected willingness to wait as $q= \mathbb {E}^{\omega }_{a}(f_{i},s_{i}) / {\Gamma }(1 + (1/p))$ where Γ denotes the gamma function. Figure 11 visualizes the resulting density functions for various choices of the age-adjusted expected willingness to wait.

### A.3.4 Patient punctuality

Patients do not always arrive on time for their scheduled appointments $b\in {\mathscr{B}}$. Instead, SiM-Care allows for patient arrivals to vary around the scheduled time $t_{b}\in \mathcal {T}$ of the appointment by including an arrival deviation. As suggested by Cayirli et al. [18], the arrival deviation from *t*_*b*_ depends on a normal distribution. We choose a mean arrival deviation of *μ* = − 5 minutes and standard deviation of *σ* = 6 minutes such that roughly 20*%* of all patients are expected to arrive late for their appointments which is consistent with the observations reported in [22].

### A.3.5 Walk-in arrivals

Walk-in patients have no prespecified time at which they are expected to arrive. Instead, SiM-Care defines for every walk-in patient an earliest arrival time $a \in \mathcal {T}$ as well as a latest arrival time $b\in \mathcal {T}$ which are both situational and thoroughly discussed in Appendix A.6. The walk-in patients’ actual arrival within the given feasible arrival interval [*a*, *b*] depends on a beta distribution. Specifically, we fit a beta distribution using maximum likelihood estimation to the empirical arrival rates reported by Wang et al. [66]. As a result, we sample the arrival times of walk-in patients from the interval [*a*, *b*] of feasible arrival times according to a beta distribution with shape parameters *p* = 1.93 and *q* = 2.94; cf. Fig. 12.

### A.3.6 Service time

SiM-Care treats the service times, i.e., the duration of treatments, as a random parameter. To sample service times, we collected a set of 21 service times in a local primary care practice. As suggested in literature [17, 67], we divide the sample into patients with and without appointment and apply a log-normal maximum likelihood fit. Histograms of our empirical samples and the resulting distributions for walk-ins and patients with appointment are depicted in Figs. 13 and 14. Based on the fitted distributions, we sample the service times of patients with appointment from a log-normal distribution with meanlog *μ* = 1.82 and sdlog *σ* = 0.692 and the service times for walk-in patients from a log-normal distribution with a meanlog *μ* = 1.254 and sdlog *σ* = 0.723. As our collected data set does not incorporate transition times, we prolong all sampled service times by one minute.

### A.3.7 Appointment cancellations

Patients that recover from all their current acute illnesses, i.e., $\mathcal {I}^{\text {act}} = \emptyset $, cancel their existing acute appointment $b^{\text {act}}\in {\mathscr{B}}$ with the age-class-specific probability *p*_*a*_ ∈ [0, 1]; compare Section 3.2.4. As long as patients suffer from acute illnesses, they only cancel their acute appointment if they require earlier treatment due to a newly emerged acute illness. All patients that have not canceled their appointment will arrive for it. As chronic illnesses are static within the model, regular appointments are never canceled.

### A.4 Key performance indicators

*Access time* measures the time a patient has to wait for an appointment, i.e, given the earliest acceptable appointment time $t\in \mathcal {T}$ and the time of the arranged appointment $t_{b}\in \mathcal {T}$ it is defined as ac-time := *t*_*b*_ − *t*.

*Access distance* measures the one-way distance patient $\rho \in \mathcal {P}$ has to travel when visiting physician $\phi \in \mathcal {G}$, i.e., tr-dist := dist(*ℓ*_*ρ*_,*ℓ*_*ϕ*_), where dist(*ℓ*_1_,*ℓ*_2_) denotes the driving distance between locations $\ell _{1} \in {\mathscr{L}}$ and $\ell _{2} \in {\mathscr{L}}$ in kilometers.

*Waiting time* measures the patient’s time spent on-site before the actual treatment commences. For walk-in patients, we define the waiting time for given walk-in arrival $t^{\text {arr}}\in \mathcal {T}$ and treatment commencement $t^{\text {treat}} \in \mathcal {T}$ as wait-time := *t*^treat^ − *t*^arr^. For patients with appointment, we define the waiting time for given time of the appointment $t_{b} \in \mathcal {T}$, patient’s arrival at the practice $t^{\text {arr}}\in \mathcal {T}$, and treatment commencement $t^{\text {treat}} \in \mathcal {T}$ as $\text {wait-time} := {\max \limits } \{ t^{\text {treat}} - \max \limits \{t_{b}, t^{\text {arr}}\} , 0 \}$.

*Utilization* describes the percentage of the physician’s available working time spent treating patients, i.e., for a session *λ* ∈Λ with total treatment duration $t\in \mathcal {T}$ it is defined as $\text {util}:= t / (\overline {o}(\lambda ) - \underline {o}(\lambda ) + \frac {1}{24})$. Note that our definition of utilization clearly underestimates a physician’s actual utilization as we do not account for additional tasks such as reporting, accounting, and answering phone calls that are not modeled in SiM-Care.

*Overtime* describes the physician’s working time beyond the anticipated buffer, i.e., if the last patient in session *λ* ∈Λ is released at time $t^{\text {rel}}\in \mathcal {T}$ it is defined as $\text {over} := \max \limits \{t^{\text {rel}} - \overline {o}(\lambda ) - \frac {1}{24}, 0 \}$; compare Fig. 3.

### A.5 Experiment initialization

At initialization, patients do not suffer from acute illnesses, i.e., $\mathcal {I}^{\text {act}}= \emptyset $ and are not considered emergencies, i.e., *ε* = 0. Furthermore, all patients are initialized without scheduled appointments, i.e., *b*^act^ = *b*^reg^ = *∅*. The consideration set of physicians $\mathcal {G}^{\text {con}} \subseteq \mathcal {G}$ per patient $\rho \in \mathcal {P}$ is determined according to Algorithm 1 where rand(*x*) for *x* > 0 denotes a uniformly distributed float from the half-closed interval [0,*x*). As a result, each patient considers all physicians within a 15 km driving radius. Physicians outside this radius are considered with a 5 % chance as some patients may choose their physician according to criteria other than proximity to their home, e.g., for historical reasons.

To initialize patients’ appointment ratings $r_{\rho }^{\text {app}} (\phi )$ and walk-in ratings $r_{\rho }^{\text {walk}} (\phi , [\lambda ])$ for every considered physician $\phi \in \mathcal {G}^{\text {con}}$ and weekly session $[\lambda ] \in {\Lambda }{/}{\sim }$, we denote the number of matches between the physician’s opening hours and patient *ρ*’s availabilities by $m(\rho , \phi ):= |\{[\lambda ]\in {\Lambda }{/}{\sim }: \alpha ([\lambda ])\land o([\lambda ])\neq \emptyset \}|$ and the maximal shortest access distance by $\text {dist}^{\max \limits }:= \max \limits _{\rho \in \mathcal {P}} \min \limits _{\phi \in \mathcal {G}} \text {dist}(\ell _{\rho }, \ell _{\phi })$. The model then initializes appointment ratings as

$$ \begin{array}{@{}rcl@{}} r_{\rho}^{\text{app}} (\phi) = \begin{cases} 3 m(\rho,\phi) - \text{dist}(\ell_{\rho},\ell_{\phi})\\ + \text{rand}(2 \text{dist}^{\max}) + 100  &\text{if } m(\rho,\phi) {>} 0\\ 0 &\text{else.} \end{cases} \end{array} $$

Walk-in ratings $r_{\rho }^{\text {walk}} (\phi , [\lambda ])$ are session specific as immediate care requires physicians to be in service. Thus, sessions in which a physician is closed are not feasible for walk-in visits which is encoded by an empty rating. The model initializes walk-in ratings as

$$ \begin{array}{@{}rcl@{}} r_{\rho}^{\text{walk}} (\phi, [\lambda]) = \begin{cases} \text{rand}(\text{dist}^{\max}) \\ - \text{dist}(\ell_{\rho},\ell_{\phi}) +100 & \text{if } o([\lambda])\neq \emptyset\\ \emptyset  &\text{else.} \end{cases} \end{array} $$

From the initialized ratings, SiM-Care subsequently determines the family physician for chronic patients as the physician from the consideration set that has the highest appointment rating, i.e., $\phi ^{\text {fam}} = \text {argmax}_{\phi \in \mathcal {G}^{\text {con}}} r_{\rho }^{\text {app}} (\phi )$ which completes the setup of all simulation entities.

Following the patients’ initialization, the global event queue $\mathcal {Q}$ is still empty and therefore running a simulation experiment would result in no agent actions. To make physicians take up their work, the model generates open- and close events *e*^opn^(*ϕ*) and *e*^clo^(*ϕ*) for every session operated by physician $\phi \in \mathcal {G}$ and adds these to $\mathcal {Q}$. To start the process of patients continuously developing acute illnesses, the model generates an initial illness event *e*^ill^(*ρ*) for every patient $\rho \in \mathcal {P}$ and adds it to $\mathcal {Q}$. Finally, to start the regular treatments of chronic illnesses ${\varsigma }\in \mathcal {I}^{\text {chro}}$, an initial follow-up event *e*^fol^(*ϕ*^fam^,*ρ*, *ς*) is generated at a randomly chosen point in time within *ς*’s follow-up interval $\nu _{{\varsigma }}\in \mathcal {T}$ according to a uniform distribution and added to $\mathcal {Q}$.

### A.6.1 Distances and travel times

SiM-Care does not feature a road network to compute travel distances and travel times. Instead, it approximates the driving distance $\text {dist} \colon {\mathscr{L}} \times {\mathscr{L}} \to \mathbb {R}$ between two locations in kilometers using the great circle distance computed through the haversine formula with a detour factor of 1.417 as determined by Boscoe et al. [9]. These authors also point out, that driving distances provide good approximations for travel times in minutes, i.e., we compute travel times by assuming a constant driving speed of 60 km/h. As a result we define the travel time $\tau \colon {\mathscr{L}} \times {\mathscr{L}} \to \mathcal {T}$ as $\tau (\ell _{1}, \ell _{2}):= \frac {\text {dist}(\ell _{1}, \ell _{2})}{60\cdot 24}$.

### A.6.2 Patients requesting appointments

Patient agents $\rho \in \mathcal {P}$ request an appointment with a physician $\phi \in \mathcal {G}$, by specifying the earliest acceptable appointment time $t\in \mathcal {T}$ and their willingness to wait for this appointment $\omega \in \mathcal {T}$. As a result, newly-arranged appointments are feasible, if and only if they are scheduled in the time interval [*t*, *t* + *ω*].

The earliest acceptable appointment time $t \in \mathcal {T}$ depends on the request. The initial treatment of acute illnesses $i\in \mathcal {I}^{\text {act}}$ is urgent, so that patients seek to schedule an appointment as soon as possible. Thus, for these initial treatments, the earliest acceptable appointment time is the time of the request $t^{\text {req}}\in \mathcal {T}$ plus a 30 minute buffer (corresponding to $\frac {1}{48}$ in decimal time) plus the direct travel time, i.e., $t= t^{\text {req}} + \frac {1}{48} + \tau (\ell _{\rho }, \ell _{\phi })$. Follow-up treatments are planned at regular intervals specified by the parameter $\nu _{i}\in \mathcal {T}$. Patients request follow-up appointments in two ways: First, at the very beginning of the follow-up interval as every patient requests a follow-up appointment directly after the treatment of illnesses that require aftercare. Second, at the very end of the follow-up interval (triggered by a follow-up event) in case no feasible appointment was available at the time of the previous treatment. In the latter case, the request is urgent and therefore the earliest acceptable appointment time is defined as above, i.e., $t= t^{\text {req}} + \frac {1}{48} + \tau (\ell _{\rho }, \ell _{\phi })$. If the follow-up appointment is requested at the beginning of the follow-up interval, the next follow-up appointment for illness $i \in \mathcal {I}$ should be scheduled after the follow-up interval has passed, i.e., we set *t* = *t*^req^ + *ν*_*i*_.

The willingness to wait $\omega \in \mathcal {T}$ defines the maximum acceptable waiting period between the earliest appointment time and the actual time of the appointment. As a result, it serves as an upper bound to the patient’s access time defined in Section 3.5. Patients’ willingness to wait for the initial treatment of acute illness $i \in \mathcal {I}^{\text {act}}$ is illness specific and given by *ω* = *ω*_*i*_. Analogously, the maximum duration chronic patients are willing to wait for their regular appointment depends on their chronic illness ${\varsigma }\in \mathcal {I}^{\text {chro}}$, i.e., *ω* = *ω*_*ς*_. If patients request a follow-up appointment for acute illness $i \in \mathcal {I}^{\text {act}}$, the willingness to wait is proportional to the length of the follow-up interval $\nu _{i}\in \mathcal {T}$. To ensure that the follow-up interval is not exceeded by an excessive time span, the willingness to wait for follow-up appointments regarding $i\in \mathcal {I}^{\text {act}}$ is $\omega = \frac {\nu _{i}}{5} + 1$. Finally, emergency patients who were denied treatment are exceptionally impatient and their willingness to wait is *ω* = 0.

Algorithm 2 describes how a patient requests an initial appointment for a newly emerged acute illness. First, patients check whether they have a pre-existing appointment within the acceptable time frame. From the patients’ point of view, pre-existing appointments are particularly convenient as they require no further actions. Therefore, patients accept pre-existing appointments as feasible, even if they exceed their willingness to wait by up to 12 hours (or $\frac {1}{2}$ in decimal time); see lines 1 and 2. If the patient’s existing appointments are infeasible for the newly emerged illness, the existing acute appointment is canceled to make room for a new, earlier, acute appointment (compare line 4 and 5).

Patients $\rho \in \mathcal {P}$ request appointments from the two currently highest rated physicians $\phi _{1}, \phi _{2} \in \mathcal {G}^{\text {con}}$ in their consideration set (compare line 7). Physicians *ϕ*_1_ and *ϕ*_2_ are queried in order of their rating, i.e., patients first request an appointment with the higher rated PCP *ϕ*_1_ and only resort to *ϕ*_2_ if the request is unsuccessful. When a physician cannot offer a fitting slot, patients reduce their rating for the respective PCP.

When their willingness to wait is at least 3 days (compare line 10), patients only accept appointments that fit their personal availability $\alpha \colon {\Lambda }{/}{\sim } \to \{0,1\}$ (cf. Section 3.2). In case *ω* ≤ 3, the request is so urgent that patients are always available.

If neither *ϕ*_1_ nor *ϕ*_2_ offer a feasible slot, the search for a feasible appointment is deemed unsuccessful and patients resort to a walk-in visit.

When patients request follow-up appointments, they mostly follow the steps outlined in Algorithm 2. The main difference concerns the inquiry process (cf. line 7 and 8), as new follow-up appointments are exclusively arranged with the physician that performed the previous treatment. Only pre-existing appointments can be used for follow-up visits although they are not with the physician that performed the previous treatment; compare line 1. If the follow-up appointment request is made at the end of the follow-up interval triggered by a follow-up event, a failure initiates a walk-in attempt to ensure the patient’s aftercare. If the follow-up appointment is requested immediately after treatment at the beginning of the follow-up interval, a failure does not lead to a walk-in attempt as the corresponding follow-up event will eventually lead to a reattempt at arranging a follow-up appointment.

Chronic patients’ regular appointments are essentially follow-up appointments and thus arranged according to the same logic. The only difference concerns the evaluation of pre-existing appointments: As regular appointments are exclusively arranged with the patient’s family physician $\phi ^{\text {fam}}\in \mathcal {G}^{\text {con}}$, pre-existing acute appointments are only perceived as feasible if they are with the family physician *ϕ*^fam^ (cf. line 1 and 2). Infeasible pre-existing acute appointments are not canceled but instead an additional regular appointment is arranged with the family physician *ϕ*^fam^ (cf. line 4 and 5). Only if the newly arranged regular appointment is before or at most 12 hours after an existing acute appointment, i.e., $t_{b^{\text {reg}}} \leq t_{b^{\text {act}}} + \frac {1}{2}$, the latter is canceled as all acute illnesses will be treated at the regular appointment.

### A.6.3 Walk-in decision making

Within SiM-Care, all walk-in visits are preceded by an unsuccessful appointment request. As walk-in visits are per se urgent, the earliest possible time $t\in \mathcal {T}$ for a walk-in visit of patient $\rho \in \mathcal {P}$ at physician $\phi \in \mathcal {G}^{\text {con}}$ is defined as the current time $t^{\text {curr}}\in \mathcal {T}$ plus a 30 minute buffer plus the direct travel time, i.e., $t= t^{\text {curr}} + \frac {1}{48} + \tau (\ell _{\rho }, \ell _{\phi })$. The patients’ willing to wait for the walk-in visit is the willingness to wait $\omega \in \mathcal {T}$ of the preceding appointment request. As a result, the patient’s walk-in visit takes place in the time interval [*t*, *t* + *ω*], unless this is impossible due to the physicians’ opening hours.

As part of the walk-in decision making, patients decide on a physician $\phi ^{*} \in \mathcal {G}^{\text {con}}$ and session *λ*^∗^∈Λ for their walk-in visit. To that end, SiM-Care computes all physician-session combinations $W \subseteq \mathcal {G}^{\text {con}} \times {\Lambda }$ that fall into the interval [*t*, *t* + *ω*] and thus can be targeted for a walk-in visit. If *W* = *∅*, the model gradually increases the willingness to wait *ω* until *W*≠*∅*.

Patients select the physician-session combination (*ϕ*^∗^,*λ*^∗^) ∈ *W* targeted for their walk-in visit on the basis of their walk-in ratings $r_{\rho }^{\text {walk}}$ via

$$ \begin{array}{@{}rcl@{}} (\phi^{*}, \lambda^{*})= \text{argmax}_{(\phi,\lambda) \in W}  0.95^{w_{t}(\lambda)} \cdot r_{\rho}^{\text{walk}} (\phi, [\lambda]), \end{array} $$

where $w_{t}(\lambda ) := \overline {o}(\lambda ) - t$ denotes the time difference between the earliest possible walk-in time $t\in \mathcal {T}$ and the end of session *λ* ∈Λ. This takes into account that walk-in patients urgently want to visit a physician by discounting the ratings based on the approximate access time *w*_*t*_(*λ*) ≥ 0. Note that this discounting model yields undesired results if we allow for negative ratings, motivating the models limitation to non-negative ratings.

Given the targeted physician-session combination (*ϕ*^∗^,*λ*^∗^) ∈ *W* for the walk-in visit, the time interval during which the actual visit at *ϕ*^∗^ may take place is defined as follows: The earliest time for walk-in patients to arrive in session *λ*^∗^∈Λ is 15 minutes before its beginning $\underline {o}(\lambda ^{*})\in \mathcal {T}$, but obviously not before the earliest possible arrival $t \in \mathcal {T}$. The latest possible arrival in session *λ*^∗^∈Λ is its ending $\overline {o}(\lambda ^{*}) \in \mathcal {T}$, but not after the latest possible arrival *t* + *ω*. The resulting time interval for the patient’s walk-in arrival is

$$ \begin{array}{@{}rcl@{}} \textstyle \left[\max(\underline{o}(\lambda^{*}) - \frac{1}{96}, t), \min(\overline{o}(\lambda^{*}), t+\omega )\right]. \end{array} $$

The patient’s actual arrival within the feasible time interval is stochastic and sampled according to the distribution specified in Section A.3.5.

As long as patients actively pursue walk-in treatment, they never arrange new appointments. That is if a walk-in patient develops a new acute illness or seeks an immediate follow-up appointment triggered by a follow-up event, their need for medical attention is met through the ongoing walk-in visit.

### A.6.2 Service time reduction

Physicians’ treatment strategies let them reduce service times to prevent congestion and minimize overtime. Within the model, the service time reduction operationalizes via a multiplicative factor *ζ* ∈ [0, 1]. Thus, a treatment with an original service time of 10 minutes (sampled from the log-normal distribution described in Section 3.4) takes only 8 minutes when performed by a physician with current consultation speed *ζ* = 0.8. When there is no effort to reduce service times, i.e., *ζ* = 1, the actual services time coincide with the sampled original service times.

### A.6.3 Consequences from rejection of patients

Whenever a patient visits a physician either with an appointment or as a walk-in, the physician’s admission strategy determines whether the patient is admitted or rejected. Following a rejection, patients reduce their personal ratings $r_{\rho }^{\text {app}}$ or $r_{\rho }^{\text {walk}}$ depending on whether they arrived for an appointment or as a walk-in. As rejected patients have been denied treatment, they are subsequently flagged as emergencies, i.e, *ε* = 1. In order to be treated, rejected patients then start a walk-in attempt with reduced willingness to wait *ω* = 0, i.e., they visit their preferred physician according to the updated walk-in preferences $r_{\rho }^{\text {walk}}$ in the earliest possible session. A patient’s emergency flag is only revoked after the next successful treatment or if the patient fully recovers from all acute illnesses.

### A.6.4 Rating adjustments

Throughout the simulation, patients adjust their ratings of physicians according to their experiences via additive factors. To that end, patients increase ratings based on positive experiences and decrease ratings following negative experiences. Thereby, patients with appointment update their appointment ratings $r_{\rho }^{\text {app}}$ while walk-in patients update their walk-in ratings $r_{\rho }^{\text {walk}}$. Table 16 lists all events that trigger a rating adjustment.

**Table 16:** Adaptation of patient ratings $r_{\rho }^{\text {app}}$ and $r_{\rho }^{\text {walk}}$

Positive Event	Adjustment
Waiting time $< 7 \min \limits $	+ 5
Successful arrangement of appointment	+ 4
Successful treatment as walk-in	+ 3*ζ*
Successful treatment with appointment	+ 2*ζ*
Negative Event	Adjustment
Waiting time $> 30 \min \limits $	− 10
No appointment within willingness available	− *ω*
Rejected as walk-in	− 10
Rejected with appointment	− 20

Parameter $\omega \in \mathcal {T}$ describes patient’s willingness to wait and *ζ* ∈ [0, 1] the physician’s consultation speed

In SiM-Care, only the effect of a failed appointment request and the effect of a successful treatment are parameterized. All other event effects are hard-coded to represent the following intuition about patient perceptions: Unanticipated events cause a stronger adjustment, while anticipated events only cause a slight adjustment. For example, visiting a physician with an appointment and not being admitted is considered highly unlikely and therefore highly penalized. Furthermore, patients react more strongly to negative experiences, reflecting the so-called *negativity bias* [8].

When a physician fails to offer a fitting appointment, the negative adjustment depends on the patient’s associated willingness to wait $\omega \in \mathcal {T}$. As *ω* ≥ 0, the adjustment − *ω* is always non-positive. When the willingness to wait is high, the expectation of receiving a fitting slot is also high, so that the resulting disappointment leads to a stronger negative adjustment.

When physicians reduce their service time as part of their treatment strategy, patients feel rushed. Therefore, the model scales the positive adjustment following a successful treatment as dependent on the physician’s current consultation speed. For example, at a consultation speed of *ζ* = 0.5 a successful treatment with appointment increases $r_{\rho }^{\text {app}}$ only by a value of 0.5 ⋅ 2 = 1.

To ensure the desired behavior of discounting ratings in the walk-in decision making, we bound all ratings from below by zero, i.e., we enforce $r_{\rho }^{\text {app}} (\phi )\geq 0$ and $r_{\rho }^{\text {walk}} (\phi , [\lambda ]) \geq 0$ for all $\rho \in \mathcal {P}$, $\phi \in \mathcal {G}$, $[\lambda ]\in {\Lambda }{/}{\sim }$. As a result, negative adjustments have no effect on physicians with a rating of zero.

### A.6.5 Family physician adjustments

Every time chronic patients adjust their appointment ratings $r_{\rho }^{\text {app}}(\phi )$ for any $\phi \in \mathcal {G}^{\text {con}}$, they simultaneously reevaluate their family physician *ϕ*^fam^ according to Algorithm 3. Thereby, chronic patients change their family physician when another physician from the consideration set has a rating that is at least 20 % higher than the current family physician’s rating.

### A.6.6 Treatment effects

Physicians treat all of a patient’s current acute illnesses $i \in \mathcal {I}^{\text {act}}$ during the same appointment. As a result, all scheduled follow-up events *e*^fol^(*ϕ*, *ρ*, *i*) for $i \in \mathcal {I}^{\text {act}}$ are deleted. Moreover, all illnesses $i\in \mathcal {I}^{\text {act}}$ that require only a single treatment, as indicated by *d*_*i*_ = *∅*, are cured and thus removed from $\mathcal {I}^{\text {act}}$. Finally, new follow-up events are scheduled for all illnesses $i \in \mathcal {I}^{\text {act}}$ that still require follow-up consultation as indicated by a positive follow-up interval *ν*_*i*_ > 0.

Chronic illnesses are only treated during the recurrent regular appointments or during walk-in visits triggered by the unavailability of a feasible regular appointment. If ${\varsigma }\in \mathcal {I}^{\text {chro}}$ is treated, any existing follow-up event $e^{\text {fol}}(\phi , \rho , {\varsigma })\in \mathcal {Q}$ is deleted and replaced by a new, updated one.

Finally, the successful treatment revokes any emergency flag the patient may have.
